# Diagnostic accuracy of low-complexity, manual nucleic acid amplification tests for the detection of pulmonary and extrapulmonary tuberculosis in adults and adolescents: a systematic review and meta-analysis∗

**DOI:** 10.1016/j.lanmic.2025.101169

**Published:** 2025-10

**Authors:** Leeberk Raja Inbaraj, Vignes Anand Srinivasalu, Mukesh Kumar Sathya Narayanan, Adhin Bhaskar, Jefferson Daniel, Katie Scandrett, Alexei Korobitsyn, Nazir Ismail, Yemisi Takwoingi

**Affiliations:** a Department of Clinical Research, Indian Council of Medical Research—National Institute for Research in Tuberculosis, Chennai, India; b Department of Epidemiology, Indian Council of Medical Research—National Institute for Research in Tuberculosis, Chennai, India; c Department of Statistics, Indian Council of Medical Research—National Institute for Research in Tuberculosis, Chennai, India; d Department of Pulmonology, Christian Medical College, Vellore, India; e Department of Applied Health Sciences, University of Birmingham, Birmingham, UK; f Global TB Program, World Health Organization, Geneva, Switzerland

## Abstract

**Background:**

Low-complexity, manual nucleic acid amplification tests, such as loop-mediated isothermal amplification for tuberculosis (TB-LAMP), are among the molecular WHO-recommended rapid diagnostics and can provide results within a few hours, even in resource-limited settings. We aimed to synthesise evidence on the accuracy of these tests for the detection of pulmonary and extrapulmonary tuberculosis, to inform the 2024 update of the WHO consolidated guidelines on tuberculosis.

**Methods:**

For this systematic review and meta-analysis, we searched the Cochrane Central Register of Controlled Trials, MEDLINE, Embase, the Science Citation Index and BIOSIS previews, WHO Global Index Medicus, and Scopus databases, for articles published from Jan 1, 1946, to Oct 2, 2023, using specific search terms such as “Tuberculosis”, “mycobacterium tuberculosis”, “pulmonary tuberculosis”, “extrapulmonary tuberculosis”, “Loopamp”, “diagnostic test”, “smear microscopy”, and “TB-LAMP”. We also examined the reference lists of the included articles to identify potentially eligible studies that were not found in the electronic searches. Additionally, we searched ClinicalTrials.gov and the WHO Clinical Trials Registry Platform for ongoing and unpublished studies. We also examined studies and data received through a WHO public call, made between Nov 30, 2023, and Feb 15, 2024, for eligibility. We included studies that evaluated design-locked, marketed technologies belonging to the class of low-complexity, manual nucleic acid amplification tests (ie, TB-LAMP) against microbiological or composite reference standards, in adults and adolescents (aged ≥10 years) with presumptive pulmonary or extrapulmonary tuberculosis. We excluded studies with case–control designs and those that used in-house methods, screening studies aimed at identifying individuals with active tuberculosis in community settings, and drug-resistance surveys. We extracted data using a standardised form and assessed risk of bias and applicability using the revised Quality Assessment of Diagnostic Accuracy Studies tool. We contacted study authors for further information and data as required. We conducted meta-analyses using bivariate random-effects models to estimate summary sensitivities and specificities for detecting pulmonary and extrapulmonary tuberculosis, and assessed the certainty of evidence using the GRADE approach. This study is registered with PROSPERO, CRD42023471548.

**Findings:**

Our searches identified 2806 records from databases and seven records from other sources. Of these, we screened the full text of 151 articles and ultimately included 29 studies in our systematic review: 27 on pulmonary tuberculosis and three on extrapulmonary tuberculosis (one study evaluated both). The studies generally had low risk of bias and applicability concern. From 26 studies involving 18 297 participants, the summary sensitivity for the detection of pulmonary tuberculosis from respiratory specimens was 84·1% (95% CI 78·3–88·6) and the summary specificity was 96·1% (95% CI 94·2–97·4), both with high certainty of evidence. Three studies, involving 95 participants, assessed the accuracy of TB-LAMP for detecting lymph node tuberculosis using lymph node tissue from biopsy. The summary sensitivity was 94·3% (79·8–98·6) and the summary specificity was 90·0% (79·5–95·4), both with low certainty of evidence.

**Interpretation:**

TB-LAMP has satisfactory performance for detecting pulmonary tuberculosis in adolescents and adults and is a potential alternative to molecular tests that require more advanced infrastructure. However, the inability to detect rifampicin resistance is an important limitation of TB-LAMP. Future research should focus on well powered studies to establish the diagnostic accuracy of TB-LAMP for extrapulmonary tuberculosis sites.

**Funding:**

WHO.

## Introduction

WHO has estimated that tuberculosis affected 10·8 million individuals and resulted in 1·25 million deaths globally in 2023.^1^ COVID-19 led to a decrease in the annual tuberculosis notification rate and an increase of 200 000 in the number of tuberculosis-related deaths between 2019 and 2021.^2^ 7·5 million people were newly diagnosed with tuberculosis in 2023 compared with 5·4 million in 2020, and the global gap between incident and notified cases persisted at 2·7 million in 2023. Underdiagnosis can lead to increased transmission of tuberculosis in the community and increased mortality. Diagnostic delays and subsequent delays in treatment initiation are primary drivers of mortality and treatment failure.

**Research in context**

**Evidence before this study**

Diagnostic delay is one of the major drivers of unfavourable treatment outcomes and mortality in individuals with tuberculosis. Low-complexity, automated nucleic acid amplification tests (NAATs), such as Xpert MTB/RIF (Cepheid, a subsidiary of Danaher, Sunnyvale, CA, USA) and Truenat MTB (Molbio Diagnostics, Bangalore, India) assays, can detect rifampicin resistance in addition to tuberculosis; however, they require laboratory infrastructure and have high operational costs. These limitations could be mitigated by low-complexity, manual NAATs, such as loop-mediated isothermal amplification for tuberculosis (TB-LAMP). This assay could be a valuable tool in the global fight against tuberculosis—particularly in regions where the disease is most prevalent and resources are scarce—owing to its cost-effectiveness, rapid turnaround time, and simplicity. A systematic review that informed the WHO policy guideline in 2016 reported moderate sensitivity and high specificity of TB-LAMP for the diagnosis of pulmonary tuberculosis in adults, and the test was subsequently recommended by WHO (conditional recommendation, very low-quality evidence). However, data among people living with HIV were scarce. A search of the Cochrane Central Register of Controlled Trials, MEDLINE, Embase, the Science Citation Index and BIOSIS previews, WHO Global Index Medicus, and Scopus databases, using specific search terms such as “Tuberculosis”, “mycobacterium tuberculosis”, “pulmonary tuberculosis”, “extrapulmonary tuberculosis”, “Loopamp”, “diagnostic test”, “smear microscopy”, and “TB-LAMP” and with no language restrictions, was carried out to identify articles published from Jan 1, 1946 to Oct 2, 2023. Among the articles retrieved, this search yielded two systematic reviews, published in 2016 and 2018; however, both included in-house assays. We therefore did a systematic review of commercially available, low-complexity, manual NAATs to inform the 2024 update of the WHO policy guideline on rapid NAATs for the detection of tuberculosis.

**Added value of this study**

We bring together available evidence on the diagnostic accuracy of low-complexity, manual NAATs in adults and adolescents (aged ≥10 years). Adhering to robust methods, we conducted a high-quality systematic review, including twice as many studies (27 studies) as in the previous review (13 studies) and more data on people living with HIV, with an increase in the certainty of evidence as assessed by the GRADE approach. TB-LAMP has high sensitivity and high specificity for the detection of pulmonary tuberculosis, with high certainty of evidence. Sensitivity was moderate (low certainty of evidence) with high specificity (moderate certainty of evidence) for the detection of pulmonary tuberculosis among people living with HIV.

**Implications of all the available evidence**

This review has informed WHO policy recommendations, including in people living with HIV, and has important implications for tuberculosis programmes globally. Considering the basic implementation requirements and reduced operational cost, TB-LAMP could be an alternative diagnostic test in resource-limited settings in which low-complexity, automated NAATs are unavailable. Future research should focus on the evaluation of TB-LAMP for extrapulmonary tuberculosis and on the analysis of alternative specimens that are easier to collect than sputum, such as stool and nasopharyngeal aspirates.

Molecular WHO-recommended rapid diagnostic tests provide early and accurate diagnosis of tuberculosis;^3^ some such tests can simultaneously detect drug resistance in addition to tuberculosis.^4^ Owing to the increasing number of these tests, WHO introduced a class-based recommendation approach in December, 2020. The three classes of test are defined by the type of technology (eg, automated or reverse hybridisation nucleic acid amplification tests [NAATs]), the complexity of the test in terms of implementation (eg, low, moderate, or high—considering the requirements of infrastructure, equipment, and technical skills of laboratory staff), and the target conditions (eg, diagnosis of tuberculosis and detection of resistance to first-line or second-line drugs).^3^ Currently, low-complexity, automated NAATs, such as Xpert MTB/RIF (Cepheid, a subsidiary of Danaher, Sunnyvale, CA, USA) and Truenat MTB (Molbio Diagnostics, Bangalore, India) assays, are widely available and are the tests of choice for the initial diagnosis of tuberculosis. However, the high cost of the equipment, cartridges, or chips and the requirement of laboratory infrastructure are barriers to uptake, particularly in low-income and middle-income countries that need them most.^3^^,^^5^ These cost and infrastructural limitations could be mitigated by low-complexity, manual NAATs, such as loop-mediated isothermal amplification for tuberculosis (TB-LAMP).

TB-LAMP is a DNA amplification technique that does not require a thermal cycler, as the DNA amplification by the loops of primers occurs at a single temperature of 65°C. The technique does not require special infrastructure and the equipment can be transported in portable vans for mobile diagnosis and operated at environmental temperatures of up to 40°C, eliminating the need for an air-conditioned room. The consumables are also cheaper than those of other molecular tests. These factors make TB-LAMP suitable for peripheral and resource-limited settings.^5^

In 2016, WHO conditionally recommended TB-LAMP as a replacement for sputum smear microscopy for diagnosing pulmonary tuberculosis in adults (aged ≥18 years) with presumptive tuberculosis. However, the test does not detect drug resistance and is best suited for individuals at low risk of drug-resistant tuberculosis and in settings in which sophisticated molecular tests are not available. This recommendation was based on a systematic review of 13 studies.^5^ Literature searches identified two systematic reviews published in 2016^6^ and 2018,^7^ but both included in-house assays. We therefore conducted this systematic review to inform the 2024 update of the WHO policy guideline on rapid NAATs for the detection of tuberculosis. We aimed to assess the diagnostic accuracy of commercially available, low-complexity, manual NAATs for the detection of pulmonary or extrapulmonary tuberculosis in adults and adolescents (aged ≥10 years) with presumptive tuberculosis.

## Methods

### Search strategy and selection criteria

For this systematic review and meta-analysis, an information specialist (Vittoria Lutje, Cochrane Infectious Diseases Group) conducted a literature search, without language restrictions and using the search terms in the appendix (pp 11–13), to identify articles published from Jan 1, 1946, to Oct 2, 2023. The databases searched were the Cochrane Central Register of Controlled Trials, included in the Cochrane Library (issue 10; October, 2023); MEDLINE (Ovid); Embase (Ovid); the Science Citation Index and BIOSIS previews (ISI Web of Knowledge); WHO Global Index Medicus; and Scopus (Elsevier). We also searched ClinicalTrials.gov and the WHO Clinical Trials Registry Platform to identify ongoing trials. Four authors (JD, MKSN, VAS, and AB) examined the reference lists of included articles and relevant review articles identified through electronic searches. The information specialist also searched for relevant dissertations in ProQuest Dissertations & Theses A&I. Two authors (LRI and MKSN) searched for information on ongoing and unpublished studies from experts working on new diagnostics for tuberculosis, such as STOP TB Partnership’s New Diagnostics Working Group and FIND, the global alliance for diagnostics. A WHO public call was made between Nov 30, 2023, and Feb 15, 2024, for ongoing and unpublished studies from manufacturers and researchers.

We included cross-sectional and cohort studies of the diagnostic accuracy of TB-LAMP for the detection of pulmonary or extrapulmonary tuberculosis. We included studies that reported the number of true positives, true negatives, false positives, and false negatives or provided statistics that enabled their derivation. We excluded studies with a two-group (ie, diagnostic case–control) design because these studies can lead to biased estimates of diagnostic accuracy.^8^ Four authors (JD, MKSN, VAS, and AB), working in two pairs, screened titles and abstracts, then reviewed the full text of potentially eligible studies. Any discrepancies between these authors were resolved by consultation with a fifth author (LRI).

We included studies that evaluated TB-LAMP in adolescents and adults (aged ≥10 years, as defined by WHO) presumed to have tuberculosis.^9^ We considered studies from all types of health facility and all laboratory levels (peripheral, intermediate, and central) from all countries. We included studies that recruited people living with HIV or diabetes or with a history of tuberculosis. We included only participants for whom the index test was conducted on respiratory samples (ie, expectorated sputum, induced sputum, bronchial alveolar lavage, or tracheal aspirates) for the evaluation of pulmonary tuberculosis. For extrapulmonary tuberculosis, we included non-respiratory specimens such as cerebrospinal fluid, pleural fluid, and lymph node aspirate or tissue. To obtain reliable estimates, we included only studies that provided at least five specimens for a given form of extrapulmonary tuberculosis. We were interested in assessing diagnostic accuracy in a passive case-finding setting in individuals with presumptive (on the basis of signs and symptoms) tuberculosis. Therefore, we excluded studies designed to find people with active tuberculosis in community settings, as these surveys are generally done for active case-finding and very often among people without symptoms. We also excluded drug-resistance surveys. If a study included children and adolescents or adults and if disaggregated data were not available in the published paper, we contacted the study authors for the data. We excluded the study if the authors did not respond or declined to provide the data or if the data were unavailable. The inclusion criteria did not differ between the systematic review and meta-analysis.

**Figure 1:**
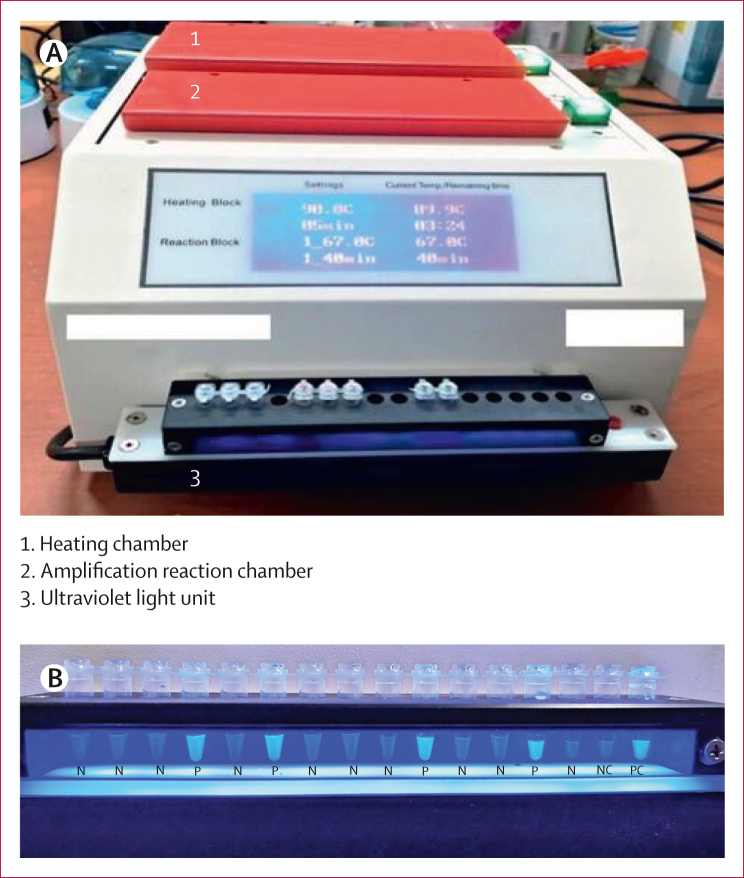
TB-LAMP setup and interpretation (A) A TB-LAMP device. (B) Visual interpretation of TB-LAMP results under ultraviolet light. LAMP=loop-mediated isothermal amplification. N=negative. NC=negative control. P=positive. PC=positive control. TB=tuberculosis.

We included only design-locked, marketed test technologies and excluded in-house methods. TB-LAMP is the only commercially available test in the class of low-complexity, manual NAATs that meets this criterion.^5^ The test involves four steps: sample transfer and lysis, DNA extraction, loop-mediated isothermal amplification, and result interpretation. How the results are interpreted differs according to the technique used. For visual-inspection methods, fluorescence indicates the presence of *Mycobacterium tuberculosis* DNA and is considered tuberculosis-positive (figure 1). TB-LAMP relies on the formation of magnesium pyrophosphate, a byproduct of DNA amplification, which causes the reaction mixture to turn turbid (cloudy). The turbidimeter detects and quantifies this turbidity in real time.^5^ The cutoff threshold for a positive result depends on the instrument settings and manufacturer guidelines and is prespecified.

We included two reference standards: culture and composite. Mycobacterial culture, using either automated liquid culture or solid culture methods, is considered the best reference standard for tuberculosis. As both culture methods are used interchangeably in clinical practice, a positive result from either solid or liquid culture alone or from both methods was accepted as a diagnosis of tuberculosis, whereas a negative culture indicated no tuberculosis.^10^ We defined a composite reference standard as a positive culture or a clinical decision to initiate treatment for tuberculosis (ie, clinically diagnosed tuberculosis). The composite reference standard could be based on the evaluation of microbiological tests, cultures, or NAATs other than the index test; imaging studies; histology; or clinical characteristics, and it should include at least one component test that is positive, according to the definition used by the authors of the primary study. Without information on tuberculosis treatment, we accepted a study-specific definition (ie, a definition of confirmed tuberculosis defined by the authors of the primary study), if available.

Detailed methods are described in the generic protocol of six diagnostic accuracy reviews that provided evidence to inform updates to the WHO policy guidelines on tests for tuberculosis detection.^11^ We reported this review in accordance with the PRISMA extension for diagnostic test accuracy studies (appendix pp 9–10).^12^ We did not obtain the approval of the institutional ethics committees as this review of secondary data was exempt.

### Data analysis

Five authors (LRI, JD, MKSN, VAS, and AB), working in pairs, independently extracted data using a standardised form and assessed study quality using the Quality Assessment of Diagnostic Accuracy Studies-2 (QUADAS-2) tool (appendix pp 14–24). Any disagreements between the authors were resolved by LRI. We extracted data such as study details (ie, setting, design, method of participant allocation, and sample size); characteristics of study participants (ie, age, sex, and HIV status); details of the index test, reference standards, and target condition; specimens (ie, number and type); and outcomes (ie, number of true positives, false positives, false negatives, and true negatives). If a study reported sample-level data or used multiple specimens, we requested participant-level data from the study authors. When such data were unavailable or if the study authors did not respond, we included the study if the number of samples exceeded the number of participants by no more than 5%, as this was considered unlikely to affect the analysis.

We conducted meta-analyses using bivariate random-effects models to estimate summary sensitivities and specificities, with 95% CIs, for pulmonary and extrapulmonary tuberculosis separately. Predictive values were calculated at a pre-test probability of 10% using the summary estimates of sensitivity and specificity, along with their 95% CIs. Our unit of analysis was per person rather than per sample. The bivariate model includes random effects that allow for between-study variation in sensitivity and specificity and a correlation parameter that allows for potential trade-off between sensitivity and specificity across studies. We fitted the models using the inbuilt meqrlogit command and the user-written metandi command in Stata version 17. Subgroup analyses were used to investigate potential sources of heterogeneity such as smear (smear microscopy positive or negative) and HIV status, and high tuberculosis burden based on the WHO classification and laboratory setting, as prespecified in the protocol (appendix p 25). Additionally, we estimated diagnostic accuracy using other respiratory and non-respiratory specimens when data were available. We conducted a sensitivity analysis by excluding studies at high or unclear risk of bias in any of the four QUADAS-2 domains.

We assessed the certainty of the evidence using the GRADE approach for reviews of diagnostic accuracy studies.^13^^,^^14^ Judgements were made separately for sensitivity and specificity. A protocol specific to this review is registered in PROSPERO (CRD42023471548; appendix pp 3–8).

### Role of the funding source

The funder established the review questions and reviewed the study protocol. The funder had no role in data collection, data analysis, data interpretation, or writing of the report.

## Results

**Figure 2:**
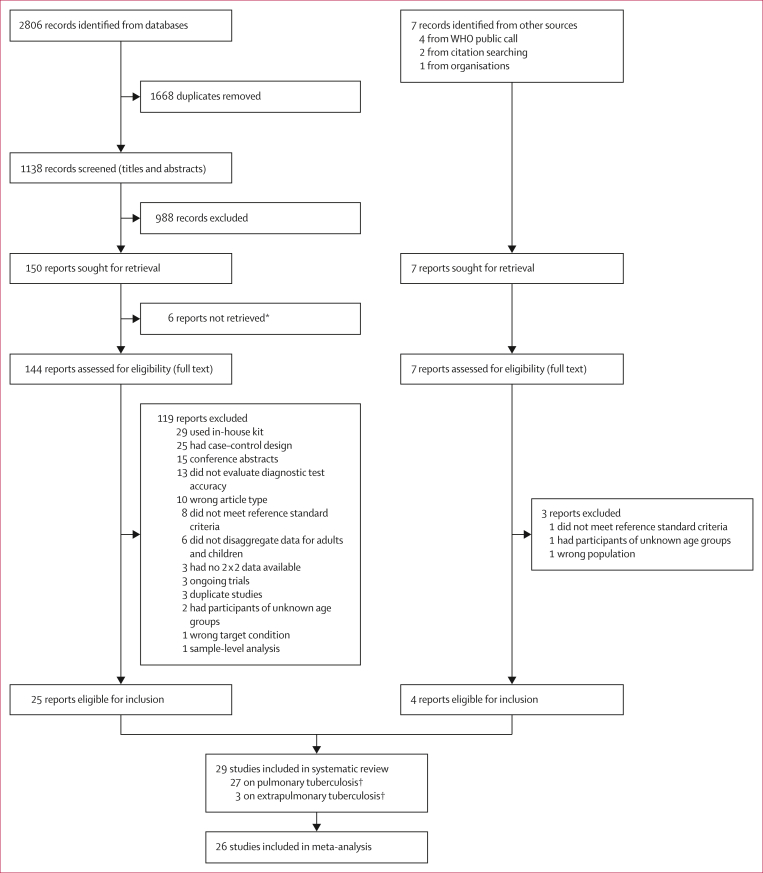
Study selection ∗These studies were most likely not eligible. †One study contributed data for both pulmonary tuberculosis and extrapulmonary tuberculosis.

We identified 2806 research articles from searches of databases and registers. After the removal of duplicates, we screened the titles and abstracts of 1138 unique articles and excluded 988 (figure 2). Seven records were identified through reference mining and the WHO public call. After report retrieval, we screened the full text of 151 articles and excluded 122 that did not meet the eligibility criteria. 29 studies were included in the systematic review,^15^, ^16^, ^17^, ^18^, ^19^, ^20^, ^21^, ^22^, ^23^, ^24^, ^25^, ^26^, ^27^, ^28^, ^29^, ^30^, ^31^, ^32^, ^33^, ^34^, ^35^, ^36^, ^37^, ^38^, ^39^, ^40^, ^41^, ^42^, ^43^ of which 27 were published^15^, ^16^, ^17^, ^18^^,^^21^, ^22^, ^23^, ^24^, ^25^, ^26^, ^27^, ^28^, ^29^, ^30^, ^31^, ^32^, ^33^, ^34^, ^35^, ^36^, ^37^, ^38^, ^39^, ^40^, ^41^, ^42^, ^43^ and two were unpublished (Donkeng Donfack et al 2024a and 2024b, Centre Pasteur du Cameroun, Cameroon, personal communication; hereafter references 19 and 20). 27 studies evaluated pulmonary tuberculosis^15^, ^16^, ^17^, ^18^^,^^20^, ^21^, ^22^, ^23^, ^24^, ^25^, ^26^, ^27^, ^28^, ^29^, ^30^, ^31^, ^32^, ^33^, ^34^, ^35^, ^36^, ^37^^,^^39^, ^40^, ^41^, ^42^, ^43^ and three evaluated extrapulmonary tuberculosis^19^^,^^35^^,^^38^ (one study^35^ evaluated both). We contacted the corresponding authors of all included studies. Of the 29 studies, five (17%) authors did not respond and one (3%) email could not be delivered. We obtained raw data for nine (31%) studies, and the authors of the remaining 14 (48%) studies either completed our data extraction form or provided additional information. The list of excluded studies with reasons for exclusion is given in the appendix (pp 26–35).

Of the 27 included studies on pulmonary tuberculosis, 18 (63%) were conducted in settings with a high tuberculosis burden (table 1). One (4%) study assessed only patients with smear-negative samples and so was not included in the main analysis for the detection of pulmonary tuberculosis.^22^ One (4%) study collected two sputum samples per participant and each sample was tested with TB-LAMP.^34^ The primary analysis reported by the study authors was based on 964 participants and 1928 sputum samples, and the participant-wise data were unavailable. We therefore extracted participant-wise data (for 976 participants) from the previously published WHO policy guidance on TB-LAMP for the diagnosis of pulmonary tuberculosis.^5^

**Table 1:** Characteristics of included studies

	Country	Reference standard	Proportion of people living with HIV	Clinical setting	Laboratory type	High tuberculosis burden	Specimen type	Proportion of smear-positive participants
Bojang et al (2016)^15^	The Gambia	MGIT	2/50 (4%)	Outpatient	Central	No	Expectorated sputum	104/300 (35%)
Cheng et al (2020)^16^	Cambodia	LJ and MGIT	3/28 (11%)	Outpatient	Peripheral	No	Expectorated sputum	115/499 (23%)
Donkeng Donfack et al (2018)^17^	Cameroon	MGIT	108/509 (21%)	Outpatient	Peripheral	No	Expectorated sputum	115/509 (23%)
Donkeng Donfack et al (2023)^18^	Cameroon	MGIT	205/354 (58%)	Outpatient	Central	No	Expectorated sputum	86/354 (24%)
Donkeng Donfack et al (2024a, personal communication)^19^∗	Cameroon	MGIT	206/928 (22%)	Outpatient	Central	No	Multiple extrapulmonary specimens (lymph node aspirate, lymph node tissue, lymph node pus, cerebrospinal fluid, pleural fluid, pleural tissue, pleural pus, ascitic fluid, synovial fluid, and urine)	48/1289 (4%)
Donkeng Donfack et al (2024b, personal communication)^20^∗	Cameroon	MGIT	1048 (100%)	Outpatient	Central	No	Expectorated sputum and bronchoalveolar lavage	Not reported
Gelaw et al (2017)^21^	Ethiopia	LJ	Not reported	Outpatient	Central	Yes	Expectorated sputum	23/78 (29%)
Getahun et al (2017)^22^†	Ethiopia	LJ or MGIT	57/190 (30%)	Outpatient	Central	Yes	Expectorated sputum	0
Gray et al (2016)^23^	India, Uganda, and Peru	LJ and MGIT	266/440 (60%)	Outpatient	Peripheral	Yes	Expectorated sputum	250/1777 (14%)
Kaku et al (2016)^24^	Haiti	MGIT	Not reported	Outpatient	Central	No	Expectorated sputum	45/209 (22%)
Kim et al (2018)^25^	South Korea	MGIT	Not reported	Outpatient	Central	Yes	Expectorated sputum	104/290 (36%)
Mitarai et al (2011)^26^	Japan	MGIT and Ogawa	Not reported	Inpatient and outpatient	Central	No	Expectorated sputum	115/160 (72%)
Nakiyingi et al (2018)^27^	Uganda	LJ and MGIT	113/233 (48%)	Outpatient	Both	Yes	Expectorated sputum	43/233 (18%)
N’guessan et al (2016)^28^	Côte d’Ivoire	MGIT	56/469 (12%)	Outpatient	Central	No	Expectorated sputum	147/469 (31%)
Nliwasa et al (2016)^29^	Malawi	LJ and MGIT	121/251 (48%)	Outpatient	Peripheral	No	Expectorated sputum	35/233 (15%)
Odume et al (2021)^30^	Nigeria	LJ	715/2636 (27%)	Outpatient	Peripheral	Yes	Expectorated sputum	75/2636 (3%)
Ou et al (2014)^31^	China	LJ	Not reported	Outpatient	Peripheral	Yes	Expectorated sputum	167/1329 (13%)
Ou et al (2016)^32^	China	MGIT	Not reported	Outpatient	Central	Yes	Expectorated sputum	187/1519 (12%)
Ou et al (2019)^33^	China	LJ	Not reported	Outpatient	Central	Yes	Expectorated sputum	267/3126 (9%)
Pham et al (2018)^34^‡	Peru, South Africa, Brazil, and Viet Nam	LJ or MGIT	96/964 (10%)	Not reported	Central	Yes	Expectorated sputum	216/964 (22%)
Promsena et al (2022)^35^∗	Thailand	MGIT and Ogawa	Not reported	Inpatient	Central	Yes	Multiple respiratory specimens (bronchoalveolar lavage, gastric aspirate, and tracheal aspirate) and lymph node tissue	4/16 (25%)
Reddy et al (2017)^36^	South Africa	MGIT	Not reported	Outpatient	Central	Yes	Expectorated sputum	52/402 (13%)
Ren et al (2023)^37^	China	MGIT	Not reported	Inpatient	Central	Yes	Expectorated sputum	126/228 (55%)
Singh et al (2021)^38^∗	India	LJ and MGIT	Not reported	Not reported	Central	Yes	Pleural fluid, ascitic fluid, cerebrospinal fluid, and lymph node tissue	Not reported
Spooner et al (2022)^39^	South Africa	MGIT	705 (100%)	Outpatient	Central	Yes	Expectorated and induced sputum	30/593 (5%)
Wahid et al (2020)^40^	Indonesia	LJ	2/29 (7%)	Outpatient	Central	Yes	Expectorated sputum	38/98 (39%)
Wang et al (2019)^41^	China	LJ	Not reported	Outpatient	Peripheral	Yes	Expectorated sputum	73/501 (15%)
Yadav et al (2017)^42^	India	MGIT	Excluded	Outpatient	Central	Yes	Expectorated sputum	37/453 (8%)
Yadav et al (2021)^43^	India	MGIT	Not reported	Outpatient	Central	Yes	Multiple respiratory specimens (expectorated sputum, bronchoalveolar lavage, gastric aspirate, and gastric lavage)	Not reported

LJ=Löwenstein–Jensen medium. MGIT=Mycobacteria Growth Indicator Tube (liquid culture).

∗ Singh et al (2021)^38^ and Donkeng Donfack et al (2024a)^19^ assessed extrapulmonary tuberculosis and Promsena et al (2022)^35^ assessed both pulmonary tuberculosis and extrapulmonary tuberculosis. Donkeng Donfack et al (2024a)^19^ and Donkeng Donfack et al (2024b)^20^ are data received by personal communication from the study authors for extrapulmonary tuberculosis and pulmonary tuberculosis, respectively; it is unclear whether the data are from the same study.

† Study included only individuals with smear-negative samples and so is included only in the subgroup analysis involving participants with smear-negative results.

‡ This was a specimen-level analysis but, owing to a small difference between the number of individuals and the specimen level 2 × 2 data, this study was included.

**Figure 3:**
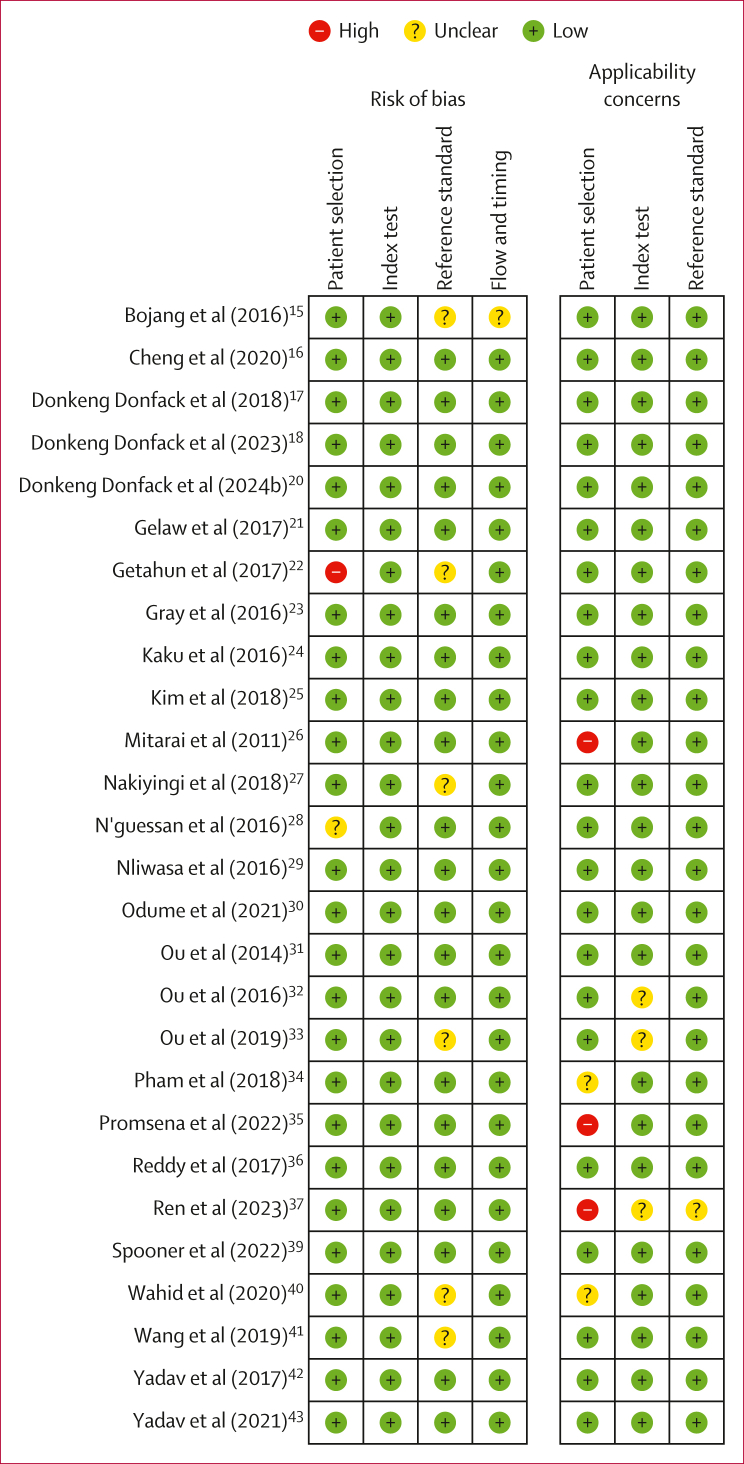
Summary of risk of bias and applicability of each study on pulmonary tuberculosis

Figure 3 summarises the risk of bias and applicability assessment of the 27 included studies for the detection of pulmonary tuberculosis. One (4%) study was judged to have a high risk of bias in the patient-selection domain, as it included only participants with smear-negative tuberculosis after triaging with smear microscopy.^22^ Similarly, we judged one (4%) study to have an unclear risk of bias in the patient-selection domain owing to lack of clarity about participant recruitment.^15^ In six (22%) studies, the reference standard was not masked and these studies were judged to have an unclear risk of bias in the reference-standard domain.^22^^,^^27^^,^^28^^,^^33^^,^^40^^,^^41^ We judged one (4%) study to have an unclear risk of bias in the flow-and-timing domain because time interval between sample collection and testing was not stated.^27^ Regarding applicability, three (11%) studies were judged to have high applicability concern in the patient-selection domain because the participants were evaluated at tertiary care centres.^26^^,^^35^^,^^37^ Three (11%) studies had unclear applicability concerns in the index-test domain owing to a lack of information on the study procedure.^28^^,^^32^^,^^37^ One (4%) study lacked information about speciation and was judged to be of unclear applicability concern in the reference-standard domain.^37^

**Figure 4:**
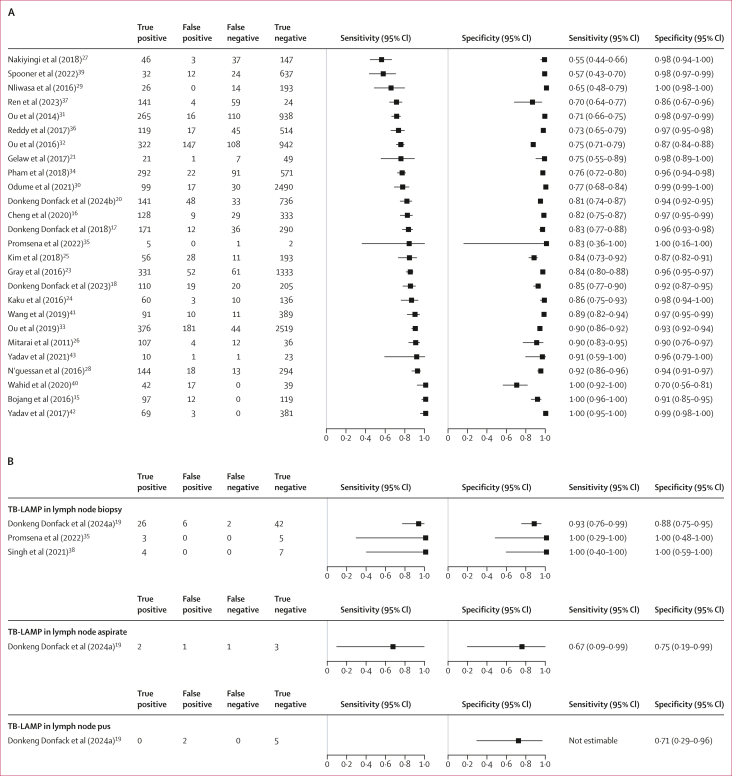
Forest plots of performance characteristics of low-complexity, manual NAATs for the detection of tuberculosis Forest plots of low-complexity, manual NAATs for the detection of pulmonary tuberculosis from respiratory samples (A) and lymph node tuberculosis (B). Studies are sorted by sensitivity, specificity, then alphabetically by author. NAATs=low-complexity manual nucleic acid amplification tests.

All 27 included studies on pulmonary tuberculosis evaluated TB-LAMP against either liquid or solid culture, and none used a composite reference standard. Therefore, all meta-analyses presented are against the microbiological reference standard. Excluding one (3%) study^22^ that included only individuals with smear-negative samples, 26 studies (involving 18 297 participants, 4108 [22·5%] of whom had pulmonary tuberculosis) were included in the meta-analysis for the detection of pulmonary tuberculosis.^15^, ^16^, ^17^, ^18^^,^^20^^,^^21^^,^^23^, ^24^, ^25^, ^26^, ^27^, ^28^, ^29^, ^30^, ^31^, ^32^, ^33^, ^34^, ^35^, ^36^, ^37^^,^^39^, ^40^, ^41^, ^42^, ^43^ The sensitivities ranged between 55% and 100%, and the specificities between 70% and 100% (figure 4A). The summary sensitivity was 84·1% (95% CI 78·3–88·6) and the summary specificity was 96·1% (95% CI 94·2–97·4; table 2; appendix p 36), both with high certainty of evidence (appendix p 41). The sensitivity analysis, excluding six studies at high or unclear risk of bias,^15^^,^^27^^,^^28^^,^^33^^,^^40^^,^^41^ gave a summary sensitivity of 80·1% (75·5–84·0) and a summary specificity of 96·7% (94·8–97·9) from 20 studies (including 13 648 participants, 3207 [23·5%] of whom had pulmonary tuberculosis).^16^, ^17^, ^18^^,^^20^^,^^21^^,^^23^, ^24^, ^25^, ^26^^,^^29^, ^30^, ^31^, ^32^^,^^34^, ^35^, ^36^, ^37^^,^^39^^,^^42^^,^^43^ Positive and negative predictive values are shown in table 2.

**Table 2:** Diagnostic accuracy of low-complexity manual NAATs for pulmonary tuberculosis in adults and adolescents

	Number of studies	Total number of participants (participants with tuberculosis [%])	Summary sensitivity (95% CI)	Summary specificity (95% CI)	Positive predictive value (95% CI)∗	Negative predictive value (95% CI)∗
**Detection of pulmonary tuberculosis**						
Sputum	26	18 297 (4108 [22·5%])	84·1 (78·3–88·6)	96·1 (94·2–97·4)	70·6 (60·0–79·1)	98·2 (97·5–98·7)
Sputum (sensitivity analysis)†	20	13 648 (3207 [23·5%])	80·1 (75·5–84·0)	96·7 (94·8–97·9)	73·0 (61·7–81·6)	97·8 (97·2–98·2)
Bronchoalveolar lavage‡	2	96 (12 [12·5%])	…	…	…	…
Gastric aspirate	1	6 (2 [33·3%])	…	…	…	…
Gastric lavage	1	24 (4 [16·7%])	…	…	…	…
**Subgroup analyses: respiratory samples**						
People living with HIV	8	2991 (460 [15·4%])	77·1 (60·8–87·9)	95·9 (84·9–99·0)	67·6 (30·9–90·7)	97·4 (95·1–98·7)
HIV-negative	3	1541 (173 [11·2%])	76·7 (57·6–88·8)	98·9 (98·2–99·3)	88·6 (78·0–93·4)	97·4 (95·4–98·8)
Smear-positive	16	1568 (1425 [90·9%])	95·9 (92·2–97·9)	43·7 (26·8–62·2)	15·9 (12·3–22·3)	99·0 (96·9–99·6)
Smear-negative	22	11 422 (1283 [11·2%])	59·9 (50·7–68·4)	97·9 (96·4–98·7)	76·0 (61·0–85·4)	95·6 (94·6–96·6)
High burden	15	11 959 (2190 [18·9%])	82·2 (72·6–89·0)	96·3 (93·2–98·0)	71·2 (54·3–83·2)	98·0 (96·8–98·8)
Central laboratory	18	10 703 (2642 [24·7%])	87·1 (79·4–92·2)	94·3 (91·4–96·3)	62·9 (50·6–73·5)	98·5 (97·6–99·1)
Peripheral laboratory	7	7484 (1402 [18·7%])	79·1 (73·1–84·0)	98·2 (96·8–99·0)	83·0 (71·7–90·3)	97·7 (97·0–98·2)

∗ Predictive values were determined at a pre-test probability of 10% using the summary estimates of sensitivity and specificity and their 95% CIs.

† This analysis excluded studies at high or unclear risk of bias. All six excluded studies—Bojang et al (2016),^15^ Nakiyingi et al (2018),^27^ N’guessan et al (2016),^28^ Ou et al (2019),^33^ Wahid et al (2020),^40^ and Wang et al (2019)^41^—had at least one domain with an unclear risk of bias, but none had a high risk of bias in any domain.

‡ A meta-analysis was not conducted for this analysis of two studies, owing to the sparse data. NAATs=nucleic acid amplification tests.

We included eight studies (involving 2991 participants, 460 [15·4%] of whom had pulmonary tuberculosis) that evaluated TB-LAMP in people living with HIV.^17^^,^^18^^,^^20^^,^^27^, ^28^, ^29^, ^30^^,^^39^ The sensitivities ranged between 52% and 100%, and the specificities between 27% and 100% (appendix p 37). The summary sensitivity was 77·1% (95% CI 60·8–87·9; low certainty of evidence) and the summary specificity was 95·9% (95% CI 84·9–99·0; moderate certainty of evidence; appendix p 42). Three studies reported estimates from HIV-negative individuals (1541 participants, 173 [11·2%] of whom had pulmonary tuberculosis).^18^^,^^29^^,^^30^ For these studies, the summary sensitivity was 76·7% (57·6–88·8) and the summary specificity was 98·9% (98·2–99·3; appendix p 37). Spooner and colleagues^39^ also reported CD4 count. Of the 690 participants with CD4 cell count data, 235 (34%) had a CD4 count of less than 200 cells per μL; the sensitivity was 60·9% (38·5–80·3; very low certainty of evidence) and the specificity was 96·2% (92·7–98·4; moderate certainty of evidence) in this population (appendix pp 37, 43).

21 studies involving 1908 participants evaluated sputum specimens from individuals with smear-positive tuberculosis,^15^^,^^17^^,^^18^^,^^21^^,^^23^, ^24^, ^25^, ^26^, ^27^, ^28^, ^29^, ^30^, ^31^, ^32^^,^^34^, ^35^, ^36^^,^^39^^,^^41^, ^42^, ^43^ although specificity could not be estimated for five studies.^23^^,^^24^^,^^29^^,^^35^^,^^43^ The sensitivities in the remaining 16 studies (involving 1568 participants, 1425 [90·9%] of whom had pulmonary tuberculosis)^15^^,^^17^^,^^18^^,^^21^^,^^25^, ^26^, ^27^, ^28^^,^^30^, ^31^, ^32^^,^^34^^,^^36^^,^^39^^,^^41^^,^^42^ that were included in the meta-analysis ranged between 64% and 100%, and the specificities between 0% and 100%. The summary sensitivity was 95·9% (95% CI 92·2–97·9) and the summary specificity was 43·7% (95% CI 26·8–62·2; appendix p 37). In individuals with smear-negative samples (22 studies^15^^,^^17^^,^^18^^,^^21^, ^22^, ^23^, ^24^, ^25^, ^26^, ^27^, ^28^, ^29^, ^30^, ^31^, ^32^^,^^34^, ^35^, ^36^^,^^39^^,^^41^, ^42^, ^43^ involving 11 422 participants, 1283 [11·2%] of whom had pulmonary tuberculosis), the summary sensitivity was 59·9% (50·7–68·4) and the summary specificity was 97·9% (96·4–98·7; appendix p 38). Sensitivity and specificity ranges for other respiratory specimens and non-respiratory specimens, such as gastric aspirate and gastric lavage, for the diagnosis of pulmonary tuberculosis are shown in the appendix (p 38). However, meta-analysis could not be conducted owing to the paucity of the data.

From seven studies conducted in peripheral laboratories,^16^^,^^17^^,^^23^^,^^29^, ^30^, ^31^^,^^41^ the summary sensitivity was 79·1% (95% CI 73·1–84·0) and the summary specificity was 98·2% (96·8–99·0). From 18 studies in central laboratories,^15^^,^^18^^,^^20^^,^^21^^,^^24^, ^25^, ^26^^,^^28^^,^^32^, ^33^, ^34^, ^35^, ^36^, ^37^^,^^39^^,^^40^^,^^42^^,^^43^ the summary sensitivity was 87·1% (79·4–92·2) and the summary specificity was 94·3% (91·4–96·3; appendix p 39).^20^ 15 studies included 11 959 participants (2190 [18·9%] of whom had pulmonary tuberculosis) from countries with a high tuberculosis burden;^21^^,^^25^^,^^27^^,^^30^, ^31^, ^32^, ^33^^,^^35^, ^36^, ^37^^,^^39^, ^40^, ^41^, ^42^, ^43^ the summary sensitivity was 82·2% (72·6–89·0) and the summary specificity was 96·3% (93·2–98·0; appendix p 39).

Three studies evaluated TB-LAMP in extrapulmonary tuberculosis specimens for the diagnosis of extrapulmonary tuberculosis. These studies were conducted in Cameroon,^19^ Thailand,^35^ and India,^38^ and all used microbiological reference standards (table 1). We judged two studies to have an unclear risk of bias as they provided no information about blinding of the reference standard to the index test results.^35^^,^^38^ One study was judged to have an unclear applicability concern in the reference-standard domain as it contained no information on speciation.^38^ Three studies (involving 95 participants, 35 of whom had lymph node tuberculosis) assessed the accuracy of TB-LAMP for detecting lymph node tuberculosis using lymph node tissue from biopsy.^19^^,^^35^^,^^39^ The estimated sensitivities were between 93% and 100%, and the specificities were between 88% and 100%. The summary sensitivity was 94·3% (95% CI 79·8–98·6; low certainty of evidence) and the summary specificity was 90·0% (95% CI 79·5–95·4; low certainty of evidence; figure 4B; appendix p 44). Two studies (involving 70 participants; three of whom had tuberculosis meningitis) assessed the accuracy of TB-LAMP for detecting tuberculosis meningitis using cerebrospinal fluid against a microbiological reference standard.^19^^,^^39^ The sensitivities were 0% (95% CI 0–84) and 100% (3–100), with very low certainty of evidence, and the specificities were 97% (95% CI 89–100) and 100% (40–100), with low certainty of evidence (appendix pp 39, 45).

Two studies (involving 67 participants, eight of whom had abdominal tuberculosis) evaluated the detection of abdominal tuberculosis using ascitic fluid.^19^^,^^39^ Estimated sensitivities were 17% (95% CI 0–64) and 100% (16–100), and estimated specificities were 94% (95% CI 73–100) and 95% (83–99; appendix p 40). Similarly, for the detection of pleural tuberculosis using pleural fluid, the estimated sensitivities from two studies (involving 292 participants, 37 of whom had pleural tuberculosis) were 48% (29–67) and 75% (35–97), and the estimated specificities were 89% (71–98) and 96% (93–98; appendix p 40).^19^^,^^39^ One study (involving five participants; one of whom had bone tuberculosis) assessed the accuracy of detecting bone or joint tuberculosis using synovial fluid.^19^ The sensitivity in this study was 100% (95% CI 3–100) and the specificity was also 100% (40–100; appendix p 40). The same study also assessed the detection of genitourinary tuberculosis using urine; the estimated sensitivity was 50% (1–99) and the specificity was 100% (88–100; appendix p 40).

## Discussion

Our systematic review included 27 studies on the detection of pulmonary tuberculosis—twice as many as in a previous review (13 studies).^44^ We were able to estimate both sensitivity and specificity for the detection of pulmonary tuberculosis with a high certainty of evidence, which makes us confident in the accuracy of TB-LAMP. The systematic review by Shete et al,^44^ which informed the previous WHO policy on TB-LAMP, reported a sensitivity of 77·7% (95% CI 71·2–83·0) and a specificity of 98·1% (95% CI 95·7–99·2) for the detection of pulmonary tuberculosis; however, we found a higher sensitivity (84·1% [78·3–88·6]) and slightly lower specificity (96·1% [94·2–97·4]).

The summary sensitivity of TB-LAMP was lower (77·1% [95% CI 60·8–87·9]) among people living with HIV than among the study population as a whole. Individuals co-infected with *M tuberculosis* and HIV usually have paucibacillary disease, with a high proportion of smear-negative tuberculosis.^45^ We found that TB-LAMP could detect approximately two-thirds of individuals who were reported as tuberculosis-negative by smear microscopy (59·9% [50·7–68·4]) when used as an add-on test after smear microscopy. The sensitivity was higher and the specificity was lower than the overall estimates among individuals with smear-positive samples. Among individuals detected as tuberculosis-positive by smear microscopy, TB-LAMP can yield false-positive results if dead bacilli or non-tuberculous mycobacteria are present, or if the samples are contaminated. In all such cases, culture and speciation will be negative. Data for evaluating extrapulmonary tuberculosis were sparse and insufficient to inform the WHO recommendations, with low to very low certainty evidence for sensitivity and specificity.

Although most of the studies included in this review had a low risk of bias, there were limitations. Some studies had high or unclear risk of bias in the patient-selection,^22^^,^^28^ reference-standard,^15^^,^^22^^,^^27^^,^^32^^,^^40^^,^^41^ or flow-and-timing^15^ domains. We also judged a few studies to have high or unclear applicability concern in the patient-selection,^26^^,^^34^^,^^35^^,^^37^^,^^40^ index-test,^31^^,^^32^^,^^37^ or reference-standard^37^ domains. However, the sensitivity analysis conducted after removing six studies^15^^,^^27^^,^^28^^,^^33^^,^^40^^,^^41^ that had either unclear or high risk of bias in one domain showed a 4% decrease in sensitivity, while the specificity remained unchanged. Our sensitivity analysis indicates that, although some studies had a high or unclear risk of bias, the quality of those studies did not significantly affect the overall results.

Our findings suggest that TB-LAMP could be an alternative test in settings in which only smear microscopy is available. TB-LAMP is suitable for use in areas with limited infrastructure, because it requires simple instrumentation that is not affected by brief power disruptions and can be operated at environmental temperatures of up to 40°C. The TB-LAMP equipment (US$2695) is less than a fifth of the price of Truenat MTB ($14 000) and Xpert MTB/RIF ($19 000) instruments.^46^ The TB-LAMP test is ideal for the detection of active cases and in settings with a high volume of samples, as it can process 14 samples in 1·5 h. However, several implementation challenges need to be considered. The disadvantages of TB-LAMP include that there is no digital output of results, which prevents the automatic transmission of results to clinicians or electronic registers, and no ability to store results in the instrument for future reference and documentation. In addition, due to the inability of TB-LAMP to detect rifampicin resistance, all positive samples need to be rapidly referred for resistance testing. TB-LAMP is also highly prone to carryover contamination when opening tubes for post-amplification analysis, which can result in false-positive results. Such contamination can be mitigated by closed-tube visual detection methods. Addressing some of these challenges through technological advancement could lead to widespread adoption of TB-LAMP.

The strengths of our review include adherence to methods recommended by the Cochrane Collaboration and extensive efforts to obtain data. Our search included a comprehensive electronic search, reference mining, a WHO public call for data, and outreach to tuberculosis experts worldwide to acquire unpublished data. We also reached out to study authors to obtain data or additional information.

In summary, TB-LAMP has high sensitivity (84·1%) and specificity (96·1%) for the detection of pulmonary tuberculosis in adults and adolescents irrespective of HIV status, and moderate sensitivity (77·1%) and high specificity (95·9%) for the detection of pulmonary tuberculosis in people living with HIV. Data were generally sparse for extrapulmonary tuberculosis. Considering the low implementation requirements and reduced operational cost, TB-LAMP could be used as an alternative diagnostic test in resource-limited settings in which low-complexity, automated NAATs are unavailable. However, the inability to detect rifampicin resistance is an important limitation. Future research should focus on the evaluation of TB-LAMP for extrapulmonary tuberculosis and non-sputum samples for the detection of pulmonary tuberculosis, such as stool, nasopharyngeal aspirate, and gastric aspirate.

## Data sharing

Data used for the analysis are available from the forest plots in the Article and from the appendix. A dataset of the same data is available upon request from the corresponding author.

## Declaration of interests

YT received funding from the WHO Global Tuberculosis Program through University of Birmingham Enterprise. All other authors declare no competing interests.
