# Supplementary material for: Diagnostic accuracy of low-complexity, manual nucleic acid amplification tests for the detection of pulmonary and extrapulmonary tuberculosis in adults and adolescents: a systematic review and meta-analysis∗
Source: Lancet Microbe. 2025 Oct;6(10):None. doi: 10.1016/j.lanmic.2025.101169 (PMC12510911; doi:10.1016/j.lanmic.2025.101169)
Supplement: Supplementary appendix [file mmc1.pdf]

THE LANCET
Microbe

Supplementary appendix

This appendix formed part of the original submission and has been peer reviewed.
We post it as supplied by the authors.

Supplement to: Inbaraj LR, Srinivasalu VA, Sathyanarayanan MK, et al. Diagnostic accuracy of low-complexity, manual nucleic acid amplification tests for the detection of pulmonary and extrapulmonary tuberculosis in adults and adolescents: a systematic review and meta-analysis. *Lancet Microbe* 2025. https://doi.org/10.1016/j.lanmic.2025.101169

Supplementary Appendix

Diagnostic accuracy of low-complexity, manual nucleic acid amplification tests for the detection of pulmonary and extrapulmonary tuberculosis in adults and adolescents: a systematic review and meta-analysis

Table of Contents

Appendix 1. Protocol registered in PROSPERO..3

Appendix 2. PRISMA DTA checklist 9

Appendix 3. Detailed search strategy. 11

Appendix 4. Quality assessment of Diagnostic accuracy (QUADAS-2) checklist for pulmonary TB..........14

Appendix 5. Quality assessment of Diagnostic accuracy (QUADAS-2) guide for extrapulmonary TB 19

Appendix 6. Additional details on statistical analysis...25

Appendix 7. Excluded studies with reason for exclusion 26

Appendix 8. Figure S1: Summary receiver characteristic operating plot of LC-mNAATs for detection of pulmonary TB in adults and adolescents 36

Appendix 9. Additional Forest plots…………………...………………………………...............................37

Figure S2: LC-mNAATs for detection of pulmonary TB in respiratory samples among PLHIV 37

Figure S3: LC-mNAATs for detection of pulmonary TB in respiratory samples among HIV negative individuals...37

Figure S4: LC-mNAATs for detection of pulmonary TB among PLHIV by CD4 count 37

Figure S5: LC-mNAATs for detection of pulmonary TB in respiratory samples among smear positive individuals...37

Figure S6: LC-mNAATs for detection of pulmonary TB in respiratory samples among smear negative individuals...38

Figure S7: LC-mNAATs for detection of pulmonary TB in bronchoalveolar lavage, gastric aspirate and gastric lavage specimens 38

Figure S8: LC-mNAATs for pulmonary TB by lab setting..39

Figure S9: LC-mNAATs for pulmonary TB in high burden setting...39

Figure S10: LC-mNAATs for detection of TB meningitis in cerebrospinal fluid...................................39

Figure S11: LC-mNAATs for detection of abdominal TB in ascitic fluid 40

Figure S12: LC-mNAATs for detection of bone or joint TB in pleural fluid.......................................40

Figure S13: LC-mNAATs for detection of bone or joint TB in synovial fluid 40

Figure S14: LC-mNAATs for detection of genitourinary TB in urine 40

Appendix 10. GRADE summary of findings tables 41

Table S1: GRADE summary of findings for LC-mNAAT on respiratory samples for detection of pulmonary TB in adults and adolescents with signs and symptoms of pulmonary TB.......................................41

Table S2: GRADE summary of findings for LC-mNAAT on respiratory samples for detection of pulmonary TB among PLHIV...42

Table S3: GRADE summary of findings for LC-mNAAT on respiratory samples for detection of pulmonary among PLHIV with CD4 <200 43

Table S4: GRADE summary of findings for LC-mNAAT on lymph node tissue for detection of lymph node TB in adults and adolescents with signs and symptoms of lymph node TB....................................44

Table S5: GRADE summary of findings for LC-mNAAT on CSF for detection of TB meningitis in adults and adolescents with signs and symptoms of TB meningitis..45

Appendix 1. Protocol registered in PROSPERO

PROSPERO

TB-LAMP (tuberculosis loop-mediated isothermal amplification) for the detection of pulmonary tuberculosis in adolescents and adults

Leeberk Raja Inbaraj, Jefferson Daniel J, Mukesh Kumar Sathyanarayanan, Vignes Anand Srinivasalu, Adhin Bhaskar, Yemisi Takwoingi

Citation

Leeberk Raja Inbaraj, Jefferson Daniel J, Mukesh Kumar Sathyanarayanan, Vignes Anand Srinivasalu, Adhin Bhaskar, Yemisi Takwoingi. TB-LAMP (tuberculosis loop-mediated isothermal amplification) for the detection of pulmonary tuberculosis in adolescents and adults. PROSPERO 2024 Available from https://www.crd.york.ac.uk/PROSPERO/view/CRD42023471548

REVIEW TITLE AND BASIC DETAILS

Review title

TB-LAMP (tuberculosis loop-mediated isothermal amplification) for the detection of pulmonary tuberculosis in adolescents and adults

Review objectives

What is the diagnostic accuracy of TB-LAMP in detecting pulmonary tuberculosis in adolescents and adults?

Index test: TB-LAMP

Reference standard: Solid or liquid culture

Outcome: Diagnostic accuracy (Sensitivity and Specificity)

Keywords

Molecular tests for TB, TB diagnostics, TB- LAMP, Tuberculosis

SEARCHING AND SCREENING

Searches

Information Specialist will perform literature searches for each index test without language restrictions. We will search the following databases using the search terms and strategy 6: Cochrane Central Register of Controlled Trials (CENTRAL), included in the Cochrane Library; MEDLINE (Ovid); Embase (Ovid); Science Citation Index and BIOSIS Previews (ISI Web of Knowledge); WHO Global Index Medicus; and Scopus (Elsevier). We will search ClinicalTrials.gov (ClinicalTrials.gov/) and the WHO Clinical Trials Registry platform (www.who.int/trialsearch) to identify ongoing trials.

Searching other resources

We will examine the reference lists of included articles and relevant review articles

identified through the electronic searches. We will identify abstracts of conferences on TB and search in ProQuest Dissertations & Theses A&I for relevant dissertations, using terms for TB and the index tests evaluated in each review. We will seek information on ongoing and unpublished studies from manufacturers through a WHO public call; and from experts working on new diagnostics for TB such as STOP TB Partnership's New Diagnostic Working Group and FIND (the global alliance for diagnostics).

Study design

We will include cross-sectional and cohort studies. We will also include randomized controlled trials that provide data on the diagnostic accuracy of an index test. We
will include only studies that report 2x2 data, i.e., the number of true positives, false positives, true negatives, and false negatives or where such data can be derived using the estimates of test accuracy reported. We will use abstracts to identify published or unpublished studies. For unpublished studies that meet the eligibility criteria, we will only include such studies if the data provided by study authors on request are the final data otherwise the studies will be documented as ongoing studies. This also includes data that may be provided by manufacturers and investigators in response to a public call for data by the WHO Global TB Programme. We will exclude case reports and diagnostic case control (two-gate) studies which are prone to bias.

ELIGIBILITY CRITERIA

Condition or domain being studied

Pulmonary tuberculosis

Population

We will include studies that evaluated the index tests for people presumed to have pulmonary tuberculosis (TB). We will consider studies from all types of health facilities and all laboratory levels (peripheral, intermediate, and central) from all countries. We will include studies that have recruited participants with HIV, diabetes mellitus, and a previous history of TB.
Based on the WHO definition of age groups, For reviews involving adults and adolescents, we will include people aged ≥10 years. We will exclude studies where we are unable to disaggregate data on adults and adolescents from those of children and studies where the age group of the participants enrolled is unknown or unclear even after requesting information from study authors. We will include participants who are untreated or who have been on TB treatment for less than 7 days. If treatment status is unclear, we will request additional information from study authors.
We will include studies that assessed the diagnostic accuracy of the index tests using expectorated or induced sputum and other respiratory specimens consistent with the intended use of the manufacturer.

Intervention(s) or exposure(s)

Our index test will be TB-LAMP (tuberculosis loop-mediated isothermal amplification).

Comparator(s) or control(s)

We do not have a comparator. We will include two reference standards: culture and composite reference standard.

Context

Tuberculosis (TB) is one of the leading causes of death worldwide and affects all ages. TB is caused by Mycobacterium tuberculosis (M. tuberculosis) bacteria. TB primarily affects the respiratory system causing pulmonary TB (PTB) but can also affect other organ systems in the form of extrapulmonary TB (EPTB). While TB is preventable and largely curable, an estimated 10.6 million people fell ill with TB worldwide in 2021 and around 1.6 million people died of TB.

The newer rapid molecular-based diagnostic methods strive to improve tuberculosis detection and reduce

diagnostic delays. Molecular WHO (World Health Organization)-recommended rapid diagnostic tests (mWRD) have better diagnostic accuracy than conventional smear microscopy. It has been recommended by the WHO to replace smear microscopy with mWRD wherever possible. One of the promising mWRD tests is TB-LAMP (tuberculosis loop-mediated isothermal amplification), a polymerase chain reaction (PCR) test that can be used as a replacement for smear microscopy in resource-limited settings because it requires the same biosafety measures and infrastructure as smear microscopy. With its rapid turnaround time, resistance to sample matrix inhibitors, high product yield, and low cost, this diagnostic tool could be a better alternative to microscopy for tuberculosis detection amongst children in low- and middle-income countries. With the availability of new studies, it is crucial that we synthesize evidence on the performance of TB-LAMP assays in adolescents and adults in order to inform the upcoming WHO guidelines.

OUTCOMES TO BE ANALYSED

Main outcomes

True positives, false positives, false negatives, true negatives, and the number of missing or unavailable test results.

Additional outcomes

None

DATA COLLECTION PROCESS

Data extraction (selection and coding)

We will design/modify a data extraction form and pilot it on at least two included studies before finalizing the form for each review. Two review authors will independently extract data and will discuss inconsistencies to achieve consensus. A third review author will be consulted if necessary. Below is a potential list of data extraction items.

1. Study details: first author; publication year; country where the study was performed; economic classification of the country according to the World Bank
2. (World Bank 2023); study setting (outpatient or inpatient setting in peripheral health
3. institutions, peripheral or intermediate laboratories); study design; method of participant allocation; total number of children screened, enrolled, excluded, and included for data analysis; study funding. We will record the stage of the study for ongoing studies (completed recruiting; completed recruitment and data cleaning; recruitment ongoing; proportion of the target sample size recruited; completed, data not yet available or published).
4. Study participants: age, HIV status, smear status, history of TB, nutritional status, household contact with TB, and previous TB treatment.
5. Target condition: PTB
6. Reference standards: number of cultures performed for each participant, either culture results alone or composite reference standard. Solid culture or automated liquid culture, or both.
7. Index tests: Details of the conduct of each assay.
8. Specimen type: number of specimens collected ; specimen type; and specimen condition
9. Outcomes: number of true positives, false positives, false negatives, true negatives, and the number of missing or unavailable test results.
10. Indeterminate and non-determinate results
11. QUADAS-2 items: information necessary for the assessment of methodological quality.
12. Other information: for example, details of industry sponsorship, if applicable.

Risk of bias (quality) assessment

We will assess methodological quality separately for each target condition in each review. Two review authors will use the QUADAS-2 tool to independently assess risk of bias and applicability concerns.

PLANNED DATA SYNTHESIS

Studies may report test accuracy per-specimen or per-person if multiple specimens of the same specimen type are taken at one or multiple timepoints for each study participant to increase diagnostic yield. Our unit of analysis is the person because per-specimen analysis may overestimate test accuracy and does not reflect clinical practice. If study authors report results per-specimen only, we will contact them to request person-level data. We will perform analyses separately for each individual index test, specimen type, target condition and reference standard (culture is the primary reference standard for TB detection). We will estimate sensitivity and specificity from each included study with 95% confidence interval (CI) and present the estimates on forest plots and in receiver operating characteristic (ROC) space on summary ROC (SROC) plots. If bivariate meta-analysis is not possible due to sparse data, or we observe little or no variability in estimates of sensitivity and specificity across studies, we will fit univariate random-effects or fixed-effect logistic regression models separately for sensitivity and specificity as appropriate (Takwoingi 2017; Takwoingi 2023). We will use the meqrlogit command in Stata 17.0 to perform meta-analysis

Analysis of subgroups or subsets

We will visually examine heterogeneity in the estimates of sensitivity and specificity for each test by assessing forest and SROC plots. If data are available, we will perform subgroup analyses or meta-regression for the following potential sources of heterogeneity.

- HIV status (negative/positive).
- Smear status (negative/positive).
- Age group: we will use WHO age groups (infant (under one year), young child (under five years), and child (under 10 years) or close to these subgroups, depending on data availability.
- History of TB (yes/no).
- Severe malnutrition in children under five years old (yes/no). We will consider Z- score below −3 standard deviations for weight-for-height, height-for-age, weight- for-age and or body mass index (BMI) to categorize as severe malnutrition.
- Laboratory setting (peripheral and intermediate laboratories): if the sample was collected in a primary healthcare facility and processed in an intermediate or reference laboratory, it will still be considered an intermediate laboratory setting.
- Healthcare setting: where study participants were recruited from for the study.
- High TB burden (yes/no): based on WHO classification (WHO 2021c).
- High TB/HIV burden (yes/no): based on WHO classification (WHO 2021c). We will perform bivariate meta-regression by including one variable at a time to assess the effect of the variable on sensitivity or specificity, or both. We will compare models with and without covariate terms for the variable using likelihood ratio tests to assess statistical significance.

REVIEW AFFILIATION, FUNDING AND PEER REVIEW

Review team members

- Dr Leeberk Raja Inbaraj, ICMR - National Institute for Research in Tuberculosis
- Dr Jefferson Daniel J, Christian Medical College, Vellore
- Dr Mukesh Kumar Sathyanarayanan, ICMR - National Institute for Research in Tuberculosis
- Dr Vignes Anand Srinivasalu, ICMR - National Institute for Research in Tuberculosis

- Dr Adhin Bhaskar, ICMR - National Institute for Research in Tuberculosis
- Professor Yemisi Takwoingi, University of Birmingham

Review affiliation

ICMR - National Institute for Research in Tuberculosis

Funding source

World Health Organization

Named contact

Leeberk Raja Inbaraj. No.1, Mayor Sathyamoorthy Road, Chethpet, Chennai, India- 600031
leeberk2003@gmail.com

TIMELINE OF THE REVIEW

Review timeline

Start date: 15 October 2023. End date: 30 April 2024

Date of first submission to PROSPERO

11 October 2023

Date of registration in PROSPERO

11 October 2023

CURRENT REVIEW STAGE

Publication of review results

The intention is to publish the review once completed. The review will be published in English.

Stage of the review at this submission

Review stage	Started	Completed
Pilot work	√	√
Formal searching/study identification	√	√
Screening search results against inclusion criteria	√	√
Data extraction or receipt of IP	√	√
Risk of bias/quality assessment	√	√
Data synthesis	√	√

Review status

The review is completed

ADDITIONAL INFORMATION

Additional information

This is a WHO commissioned review. Our findings will inform Guideline Development group that will convene in 2024 and recommend guidelines on "Molecular methods for TB diagnosis:

PROSPERO version history

- Version 1.0 published on 11 Oct 2023

Review conflict of interest

None known

Country

England, India

Medical Subject Headings

Adolescent; Adult; Humans; Reference Standards; Sensitivity and Specificity; Tuberculosis; Tuberculosis, Pulmonary

Appendix 2. PRISMA DTA checklist

Section/topic	#	PRISMA-DTA Checklist Item	Reported on page #
**TITLE / ABSTRACT**			
Title	1	Identify the report as a systematic review (+/- meta-analysis) of diagnostic test accuracy (DTA) studies.	1
Abstract	2	Abstract: See PRISMA-DTA for abstracts.	3
**INTRODUCTION**			
Rationale	3	Describe the rationale for the review in the context of what is already known.	4
Clinical role of index test	D1	State the scientific and clinical background, including the intended use and clinical role of the index test, and if applicable, the rationale for minimally acceptable test accuracy (or minimum difference in accuracy for comparative design).	4
Objectives	4	Provide an explicit statement of question(s) being addressed in terms of participants, index test(s), and target condition(s).	5
**METHODS**			
Protocol and registration	5	Indicate if a review protocol exists, if and where it can be accessed (e.g., Web address), and, if available, provide registration information including registration number.	5
Eligibility criteria	6	Specify study characteristics (participants, setting, index test(s), reference standard(s), target condition(s), and study design) and report characteristics (e.g., years considered, language, publication status) used as criteria for eligibility, giving rationale.	6
Information sources	7	Describe all information sources (e.g., databases with dates of coverage, contact with study authors to identify additional studies) in the search and date last searched.	5
Search	8	Present full search strategies for all electronic databases and other sources searched, including any limits used, such that they could be repeated.	5
Study selection	9	State the process for selecting studies (i.e., screening, eligibility, included in systematic review, and, if applicable, included in the meta-analysis).	7
Data collection process	10	Describe method of data extraction from reports (e.g., piloted forms, independently, in duplicate) and any processes for obtaining and confirming data from investigators.	7
Definitions for data extraction	11	Provide definitions used in data extraction and classifications of target condition(s), index test(s), reference standard(s) and other characteristics (e.g. study design, clinical setting).	7
Risk of bias and applicability	12	Describe methods used for assessing risk of bias in individual studies and concerns regarding the applicability to the review question.	7
Diagnostic accuracy measures	13	State the principal diagnostic accuracy measure(s) reported (e.g. sensitivity, specificity) and state the unit of assessment (e.g. per-patient, per-lesion).	7

Synthesis of results	14	Describe methods of handling data, combining results of studies and describing variability between studies. This could include, but is not limited to: a) handling of multiple definitions of target condition. b) handling of multiple thresholds of test positivity, c) handling multiple index test readers, d) handling of indeterminate test results, e) grouping and comparing tests, f) handling of different reference standards	
Meta-analysis	D2	Report the statistical methods used for meta-analyses, if performed.	7
Additional analyses	16	Describe methods of additional analyses (e.g., sensitivity or subgroup analyses, meta-regression), if done, indicating which were pre-specified.	7
**RESULTS**			
Study selection	17	Provide numbers of studies screened, assessed for eligibility, included in the review (and included in meta-analysis, if applicable) with reasons for exclusions at each stage, ideally with a flow diagram.	8
Study characteristics	18	For each included study provide citations and present key characteristics including: a) participant characteristics (presentation, prior testing), b) clinical setting, c) study design, d) target condition definition, e) index test, f) reference standard, g) sample size, h) funding sources	9 (Table.1)
Risk of bias and applicability	19	Present evaluation of risk of bias and concerns regarding applicability for each study.	7 (Fig.2)
Results of individual studies	20	For each analysis in each study (e.g. unique combination of index test, reference standard, and positivity threshold) report 2x2 data (TP, FP, FN, TN) with estimates of diagnostic accuracy and confidence intervals, ideally with a forest or receiver operator characteristic (ROC) plot.	Fig.3
Synthesis of results	21	Describe test accuracy, including variability; if meta-analysis was done, include results and confidence intervals.	9
Additional analysis	23	Give results of additional analyses, if done (e.g., sensitivity or subgroup analyses, meta-regression; analysis of index test: failure rates, proportion of inconclusive results, adverse events).	9-11
**DISCUSSION**			
Summary of evidence	24	Summarize the main findings including the strength of evidence.	11
Limitations	25	Discuss limitations from included studies (e.g. risk of bias and concerns regarding applicability) and from the review process (e.g. incomplete retrieval of identified research).	12
Conclusions	26	Provide a general interpretation of the results in the context of other evidence. Discuss implications for future research and clinical practice (e.g. the intended use and clinical role of the index test).	12
**FUNDING**			
Funding	27	For the systematic review, describe the sources of funding and other support and the role of the funders.	13

Adapted From: McInnes MDF, Moher D, Thombs BD, McGrath TA, Bossuyt PM, The PRISMA-DTA Group (2018). Preferred Reporting Items for a Systematic Review and Meta-analysis of Diagnostic Test Accuracy Studies: The PRISMA-DTA Statement. JAMA. 2018 Jan 23;319(4):388-396. doi: 10.1001/jama.2017.191

Appendix 3. Detailed search strategy

Ovid MEDLINE(R) ALL <1946 to September 29, 2023>

1 Mycobacterium tuberculosis/

2 Tuberculosis, Pulmonary/

3 Extensively Drug-Resistant Tuberculosis/

4 Tuberculosis, Multidrug-Resistant/

5 (tuberculosis or TB).tw.

6 ((extrapulmonary or lymph node* or mening* or pulmonary) and TB).ti. or ((extrapulmonary or lymph node* or mening* or pulmonary) and TB).ab.

7 exp Tuberculosis, Extrapulmonary/

8 5 and 6

9 7 or 8

10 1 or 2 or 3 or 4 or 9

11 TB-LAMP*.mp.

12 Loop-Mediated Isothermal Amplification.mp.

13 LAMP.mp.

14 LOOPAMP*.mp.

15 12 or 13 or 14

16 10 and 15

17 11 or 16

Embase 1947-Present, updated daily

1 Mycobacterium tuberculosis/

2 Lung tuberculosis/

3 extensively drug resistant tuberculosis/

4 drug resistant tuberculosis/

5 (tuberculosis or TB).tw.

6 ((extrapulmonary or lymph node* or mening* or pulmonary) and TB).ti. or ((extrapulmonary or lymph node* or mening* or pulmonary) and TB).ab.

7 extrapulmonary tuberculosis/

8 5 and 6

9 7 or 8

10 1 or 2 or 3 or 4 or 9

11 TB-LAMP*.mp.

12 Loop-Mediated Isothermal Amplification.mp.

13 LAMP.mp.

14 LOOPAMP*.mp.

15 12 or 13 or 14

16 10 and 15

17 11 or 16

Web of Science Search Strategy (Science Citation index-Expanded and Biosis previews)

#1 Search: tuberculosis or TB (Topic)

#2 Search: (extrapulmonary or lymph node* or mening* or pulmonary or lung) (Topic)

#3 Search: multidrug resistant tuberculosis or extensively drug resistant tuberculosis (Topic)

#4 Search: MDR-TB or XDR-TB (Topic)

#5 Search: #1 AND #2

#6 Search: #3 OR #4 OR #5

#7 Search: Loop-Mediated Isothermal Amplification (Topic)

#8 Search: TB-LAMP* or LOOPAMP* (Topic)

#9 Search: #7 OR #8

#10 Search: #6 AND #9

Scopus Elsevier

((TITLE-ABS-KEY (loop-mediated AND isothermal AND amplification)) OR (TITLE-ABS-KEY (loop AND mediated AND isothermal AND amplification)) OR (TITLE-ABS-KEY (tb-lamp* OR loopamp*))) AND ((TITLE-ABS-KEY ((tuberculosis OR tb) AND (extrapulmonary OR lymph AND node* OR mening* OR pulmonary))) OR (TITLE-ABS-KEY ((multidrug AND resistant AND tuberculosis) OR (extensively AND drug AND resistant AND tuberculosis))) OR (TITLE-ABS-KEY (mdr-tb OR xdr-tb)))

ProQuest Dissertations & Theses A&I

Tuberculosis and Loop-Mediated Isothermal Amplification and (diagnos* or detect* or assay*) OR TB-LAMP

Search Name: Cochrane Central Register of Controlled Trials

Issue 10 of 12, October 2023

#1 MeSH descriptor: [Tuberculosis] explode all trees

#2 MeSH descriptor: [Mycobacterium tuberculosis] explode all trees

#3 ((tuberculosis or TB or MDR-TB or XDR-TB)):ti,ab,kw

#4 #1 or #2 or #3

#5 extrapulmonary tuberculosis

#6 MeSH descriptor: [Tuberculosis, Extrapulmonary] explode all trees

#7 PTB or EPTB

#8 #1 or #2 or #3 or #4 or #5 or #6 or #7

#9 Loop-Mediated Isothermal Amplification

#10 (TB LAMP or LAMP or LOOPAMP):ti,ab,kw

#11 #9 or #10

#12 #8 and #11

Clinicaltrials.gov

Loop-Mediated Isothermal Amplification | Tuberculosis

TB-LAMP | Tuberculosis

WHO ICTRP

tuberculosis and (LAMP or TB-LAMP)

Global Index Medicus

tw:((tw:(tb-lamp)) OR (tw:((loop-mediated isothermal amplification) AND tuberculosis)

Appendix 4. Quality assessment of Diagnostic accuracy (QUADAS-2) checklist for pulmonary TB

Risk of bias judgement

For the judgement regarding risk of bias for all the domains, if only one signalling question was answered ‘no’ or ‘unclear’, we discussed further before making the risk of bias judgment for the domain. We judged ‘low’ if all signalling questions were answered ‘yes’. We judged ‘high’ if all or most signalling questions were answered ‘no’. We judged ‘unclear’ if all or most signalling questions were answered unclear.

Summary of the protocol:

Participants	• Adults and adolescents aged ≥10 years who are presumed to have pulmonary tuberculosis<br>• Include HIV, diabetes mellitus, and a previous history of TB.
Setting	• All types of health facilities and all laboratory levels (peripheral, intermediate, and central) from all countries.
Specimen	• Expectorated or induced sputum or bronchial alveolar lavage or tracheal aspiration, or tongue swabs
Exclusion	• Took ATT in the past seven days<br>• Already on ATT for the current illness
Condition	Pulmonary TB
Index test	LC-mNAATs: TB-LAMP
Reference standard:	1. **Culture:** either automated liquid culture or solid culture methods<br>2. **Composite reference standard:** either a positive culture or NAAT other than TB-LAMP or radiology or histopathology a clinical diagnosis based on clinical criteria / clinical scoring system.

Signalling question	Rationale for judgement
**1. Participant selection**	
1.1 Was a consecutive or random sample of patients enrolled?	**Yes**: if enrolment was either consecutive or random.<br><br>**No**: if it is clear that a different selection procedure was used, e.g. convenience sampling.<br><br>**Unclear**: if the selection procedure was unclear or not reported.
1.2 Was a case-control design avoided?	All participants should come from the same group of patients with presumptive TB. We answered 'Yes' for all studies by default exclude case-control designs.<br><br>**Yes**: By default.
1.3 Did the study avoid inappropriate exclusions?	We expected studies to include a representative population with presumptive TB that may include people who are treatment-naïve and who have previously received treatment for TB, irrespective of sputum smear status or the result of other related investigations.<br><br>**Yes**: if the study included a representative sample of patients, including both smear-positive and smear-negative individuals.<br><br>**No**: if the study included primarily or exclusively smear-positive or smear-negative patients or if the study included primarily or exclusively patients who had undergone previous treatment (retreatment patients).<br><br>**Unclear**: if we were unable to make a judgement of Yes or No based on the available information.
Could the selection of participants have introduced bias?	If only one signalling question was answered No or Unclear, we discussed further before making the risk of bias judgement for the domain.<br><br>**Low**: if all signalling questions were answered Yes.<br><br>**High**: if all or most signalling questions were answered No.<br><br>**Unclear**: if all or most signalling questions were answered Unclear.
1.4 Were there concerns that the included patients and setting do not match the review question?	We were interested in how the index test was performed in adults and adolescents who were evaluated for TB as they would be in routine practice.<br><br>**Low**: if patients were evaluated in local hospitals, community or primary care centres or if the sample was collected at a peripheral centre but processed in a tertiary laboratory.<br><br>**High**: if patients were evaluated exclusively as inpatients in tertiary care centres or medical colleges, or if the specimens were from stored samples in a central laboratory, or if the setting did not match the review question, for example using the index for decisions about the need for airborne isolation.<br><br>**Unclear**: if the clinical setting was not reported or the information available is insufficient to make a judgement. We also answered Unclear if the index test was done at a central-level laboratory, and the clinical setting was not reported for the following reason: it is difficult to tell if a given reference laboratory provided services mainly to very sick patients (inpatients in tertiary care) or to all patients, including very sick patients and those with less severe disease (primary, secondary, and tertiary care).
**2. Index test**	
2.1 Were the index test results interpreted without knowledge	**Yes**: We answered 'yes' if TB-LAMP was done and interpreted by different people without the knowledge of the results of the reference standards or if TB LAMP and the reference standards were done in different labs. Since

Signalling question	Rationale for judgement
of results of the reference standard?	clinical diagnosis is part of the reference standard, if the TB-LAMP interpretation and the clinical diagnosis is done by the same person, there will be bias.<br><br>**No**: if the index test interpreter is aware of the clinical history of the participant or the results of the reference standard or other index tests.<br><br>**Unclear**: If we were unable to make a judgement of Yes or No.
2.2 If a threshold was used, was it pre-specified?	**Yes**: Predefined for TB-LAMP<br><br>**Unclear**: If there was no proper description
Could the conduct or interpretation of the index test have introduced bias?	If only one signalling question was answered No or Unclear, we discussed further before making the risk of bias judgement for the domain.<br><br>**Low**: if all signalling questions were answered Yes.<br><br>**High**: if all or most signalling questions were answered No.<br><br>**Unclear**: if all or most signalling questions were answered Unclear.
2.3 Are there concerns that the index test, its conduct, or its interpretation differs from the review question?	**Low**: if the index test was performed as recommended by the manufacturer. If a particular study evaluated different types of specimens such sputum, BAL, tongue swab, tracheal aspirate etc, we used the following rule: if ≥ 75% of the specimen types were processed per the manufacturer's instructions, then we judged as Low Concern.<br><br>**High**: if the persons administering and interpreting the test clearly did not follow the manufacturer's instructions.<br><br>In case of multiple types of specimens, if < 50% of the specimen types were processed according to the book or as per the manufacturer's instructions, we judged as High Concern.<br><br>**Unclear**: if the description of the test processes was insufficient to make a Yes or No judgement.<br><br>If a study evaluated several different types of specimens, if at least 50% to 74% of the specimen types were processed according to the manufacturer's instructions, or if we could not tell, we judged as Unclear Concern.
**3A. Reference standard - CULTURE**	
3.1A Is the reference standard likely to correctly classify the target condition?	**Yes**: if a study used any of the solid or automated liquid culture methods, or a combination of these methods.<br><br>**No**: if the study did not use culture methods or a clinical composite reference standard.<br><br>**Unclear**: If we were unable to make a judgement of YES or NO.
3.2A Were the reference standard results interpreted without knowledge of results of the index test?	**Yes**: if the reference standard provided an automated result (e.g. MGIT 960), blinding was explicitly stated, or it was clear that the reference standard was performed at a separate laboratory and/or performed by different people.<br><br>**No**: if the study stated that the reference standard result was interpreted with knowledge of the index test result.<br><br>**Unclear**: If we were unable to make a judgement of Yes or No based on the available information.

Signalling question	Rationale for judgement
Could the reference standard, its conduct, or its interpretation have introduced bias?	If only one signalling question was answered No or Unclear, we discussed further before making the risk of bias judgement for the domain.<br><br>**Low**: if all signalling questions were answered Yes.<br><br>**High**: if all or most signalling questions were answered No.<br><br>**Unclear**: if all or most signalling questions were answered Unclear.
3.3AAre there concerns that the target condition as defined by the reference standard does not match the question?	**Low**: if the study performed speciation.<br><br>**High**: if the study did not speciate mycobacteria isolated in culture.<br><br>**Unclear**: if we were unable to make a judgement of Low or High CONCERN based on the available information.
**3B. Reference standard – COMPOSITE REFERENCE STANDARD**	
3.1B Is the reference standard likely to correctly classify the target condition?	The purpose of a composite reference standard is to diagnose people in whom TB was not detected by culture. The composite reference standard could be defined differently in each study. We identified people as having TB regardless of the criteria used in a study; study-specific criteria for defining TB were accepted. A key criterion is the initiation of TB treatment following a diagnosis of TB.<br><br>**No**: if the study did not use culture methods or a clinical composite reference standard.<br><br>**Unclear**: If we were unable to make a judgement of Yes or No. For children, also if studies used only one specimen.<br><br>**For composite reference standard, always judge as "Unclear".**
3.2B Were the reference standard results interpreted without knowledge of results of the index test?	**Yes**: if the reference standard was performed at a separate laboratory and/or performed by different people.<br><br>**No**: if the study stated that the reference standard result was interpreted with knowledge of the index test result.<br><br>**Unclear**: If we were unable to make a judgement of Yes or No based on the available information.
Could the reference standard, its conduct, or its interpretation have introduced bias?	If only one signalling question was answered No or Unclear, we discussed it further before making the risk of biased judgment for the domain.<br><br>**Low**: if all signalling questions were answered Yes.<br><br>**High**: if all or most signalling questions were answered No.<br><br>**Unclear**: if all or most signalling questions were answered Unclear.
Are there concerns that the target condition as defined by the reference standard does not match the question?	**Low**: if treatment was initiated after diagnosing using the composite reference standard<br><br>**HIGH**: if treatment was not initiated after using the CRS<br><br>**Unclear**: if we were unable to make a judgement of Low or High concern based on the available information.
**4. Flow and timing**	
4.1 Was there an appropriate interval between the index test and the reference standard?	**Yes**: if the index test and reference standard were performed at the same time, or if the time interval was less than or equal to seven days.<br><br>**No**: if the time interval was greater than seven days

Signalling question	Rationale for judgement
	**Unclear**: If we were unable to make a judgement of Yes or No based on the available information.
4.2 Did all patients receive the same reference standard?	**Yes**: if all participants in the study or a subset of participants in the study (for whom we extracted data) received the acceptable reference standard either culture or a composite reference standard. Regarding culture, we acknowledge that it is possible that some specimens could undergo solid culture and others liquid culture as the reference standard.<br><br>**No**: if not all participants received the same reference standard<br><br>**Unclear**: If we were unable to make a judgement of Yes or No.
4.3 Were all patients included in the analysis?	**Yes**: if the number of participants enrolled was clearly stated and correspond to the number presented in the analysis or if exclusions were adequately described.<br><br>**No**: if there were participants missing or excluded from the analysis and no explanation was given.<br><br>**Unclear**: If we were unable to make a judgement of Yes or No based on the available information.
Could the patient flow have introduced bias?	If only one signalling question was answered NO or UNCLEAR, we discussed further before making the risk of bias judgement for the domain.<br><br>**Low**: if all signalling questions were answered Yes.<br><br>**High**: if all or most signalling questions were answered No.<br><br>**Unclear**: if all or most signalling questions were answered Unclear.

Appendix 5. Quality assessment of Diagnostic accuracy (QUADAS-2) guide for extrapulmonary TB

Risk of bias judgement

For the judgement regarding risk of bias for all the domains, if only one signalling question was answered 'no' or 'unclear', we discussed further before making the risk of bias judgment for the domain. We judged 'low' if all signalling questions were answered 'yes'. We judged 'high' if all or most signalling questions were answered 'no'. We judged 'unclear' if all or most signalling questions were answered unclear.

Our protocol summary for reference:

Participants	• Adults and adolescents aged ≥10 years who are presumed to have extrapulmonary TB<br>• Include HIV, diabetes mellitus, and a previous history of TB.
Setting	• All types of health facilities and all laboratory levels (peripheral, intermediate, and central) from all countries.
Specimens	• CSF – Tuberculous meningitis<br>• Pleural fluid – Pleural TB<br>• Lymph node aspirate – Lymph node TB<br>• Joint fluid – Bone or Joint TB<br>• Urine – Genitourinary TB<br>• Pericardial fluid – Pericardial Tb<br>• Blood - disseminated TB<br>• Tissue – Any of the extrapulmonary TB<br>• We anticipate many small studies and a range of specimen types therefore we included studies that provide **at least five specimens for a given form** of extrapulmonary TB.
Exclusion	• Took ATT in the past seven days.<br>• Already on ATT for the current illness.<br>• To avoid selection bias, we excluded studies that use an index test to diagnose relapse of previously treated extrapulmonary TB since such participants will have a higher risk of extrapulmonary TB.
Condition	• Tuberculous meningitis<br>• Pleural TB<br>• Lymph node TB<br>• Genitourinary TB<br>• Bone or joint TB<br>• Peritoneal TB<br>• Pericardial TB<br>• Disseminated TB
Index test	LC-mNAATs: TB-LAMP
Reference standard:	1. **Culture:** either automated liquid culture or solid culture methods

	2. **Composite reference standard:** either a positive culture or NAAT other than TB-LAMP or radiology or histopathology a clinical diagnosis based on clinical criteria / clinical scoring system.

QUADAS-2 Guide

**Signalling question**	**Rationale for judgement**
**1. Participant selection**	
1.1 Was a consecutive or random sample of patients enrolled?	**Yes**: if enrolment was either consecutive or random.<br><br>**No**: if it is clear that a different selection procedure was used, e.g. convenience sampling.<br><br>**Unclear**: if the selection procedure was unclear or not reported.
1.2 Was a case-control design avoided?	All participants should come from the same group of patients with presumptive TB. We answered 'yes' for all studies by default exclude case-control designs.<br><br>**Yes**: By default.
1.3 Did the study avoid inappropriate exclusions?	**Yes**: if the study included a representative sample of patients, irrespective of smear-status. However, in case of extrapulmonary TB, it was acceptable if the study included only smear-negative individuals – we still gave low.<br><br>**No**: if the study included primarily or exclusively patients who had undergone previous treatment (retreatment patients) or if the study excluded specimens based on physical appearance (such as purulence) or a biochemical analysis (e.g. adenosine deaminase (ADA), cytology (cell analysis)).<br><br>**Unclear**: if we were unable to make a judgement of Yes or No based on the available information.
Could the selection of participants have introduced bias?	If only one signalling question was answered No or Unclear, we discussed further before making the risk of bias judgement for the domain.<br><br>**Low**: if all signalling questions were answered Yes.<br><br>**High**: if all or most signalling questions were answered No.<br><br>**Unclear**: if all or most signalling questions were answered Unclear.
Are there concerns that the included patients and setting do not match the review question?	**Low**: We expected patients to be evaluated in tertiary care settings for most extrapulmonary TB so default answer is low (unless stored samples were used).<br><br>**High**: if the specimens were from stored samples in a central laboratory
**2. Index test**	
2.1 Were the index test results interpreted without knowledge of results of the reference standard?	**Yes**: We answered 'Yes' if TB-LAMP was done and interpreted by different people without the knowledge of the results of the reference standards or if TB LAMP and the reference standards were done in different labs. Since clinical diagnosis is part of the reference standard, if the TB-LAMP interpretation and the clinical diagnosis is done by the same person, there will be bias.<br><br>**No**: if the index test interpreter is aware of the clinical history of the participant or the results of the reference standard or other index tests.<br><br>**Unclear**: If we were unable to make a judgement of Yes or No.

Signalling question	Rationale for judgement
2.2 If a threshold was used, was it pre-specified?	**Yes**: Predefined for TB-LAMP<br><br>**Unclear**: If there was no proper description
Could the conduct or interpretation of the index test have introduced bias?	If only one signalling question was answered No or Unclear, we discussed further before making the risk of bias judgement for the domain.<br><br>**Low**: if all signalling questions were answered Yes.<br><br>**High**: if all or most signalling questions were answered No.<br><br>**Unclear**: if all or most signalling questions were answered Unclear.
Are there concerns that the index test, its conduct, or its interpretation differs from the review question?	**Low**: if TB-LAMP was performed according to the WHO operational handbook on TB (WHO 2021a), or if TB-LAMP was performed as recommended by the manufacturer. If a particular study evaluated different types of specimens such pleural fluid, lymph node aspirate, urine etc., we would use the following rule: if ≥ 75% of the specimen types were processed per WHO handbook or as per the manufacturer's instructions, then we judged as low concern*<br><br>**High**: if the persons administering and interpreting TB-LAMP clearly did not follow the WHO handbook or the manufacturer's instructions.<br><br>In case of multiple types of specimens, if < 50% of the specimen types were processed according to the WHO handbook **or** as per the manufacturer's instructions, we judged as HIGH CONCERN.<br><br>**Unclear**: if the description of the test processes was insufficient to make a Yes or No judgement **or** if the study evaluated several different types of specimens and at least 50% to 74% of the specimen types were processed according to the WHO handbook or as per the manufacturer's instructions, or if we could not tell, we judged as unclear concern.<br><br>*See appendix below
**3A. Reference standard - CULTURE**	
3.1A Is the reference standard likely to correctly classify the target condition?	Bacterial load is usually low in extrapulmonary TB, leading to a reduction in the sensitivity of culture. Concerning the conduct of the reference standard (preparation of the specimen for culture), N-acetyl-L-cysteine-sodium hydroxide is routinely used to homogenize, decontaminate, and liquefy non-sterile specimens for TB culture (American Thoracic Society 2000). However, CSF, pleural fluid, and lymph node aspirates are usually considered sterile, and standards specify, "specimens collected from normally sterile sites may be placed directly into the culture medium" (American Thoracic Society 2000). Overly processing (sterile) specimens with N-acetyl-L-cysteine-sodium hydroxide may lead to a decrease in viable TB bacteria and consequently false-negative cultures.<br><br>**Yes**: if a study used any of the solid or automated liquid culture methods, or a combination of these methods. In addition, for extrapulmonary TB, studies should not use N-acetyl-L-cysteine-sodium hydroxide for processing specimens.<br><br>**No**: if the study did not use culture methods or a clinical composite reference standard.<br><br>**Unclear**: If we were unable to make a judgement of Yes or No or if studies used N-acetyl-L-cysteine-sodium hydroxide.

Signalling question	Rationale for judgement
3.2A Were the reference standard results interpreted without knowledge of results of the index test?	**Yes**: if the reference standard provided an automated result (e.g. Mgit 960), blinding was explicitly stated, or it was clear that the reference standard was performed at a separate laboratory and/or performed by different people.<br>**No**: if the study stated that the reference standard result was interpreted with knowledge of the index test result.<br>**Unclear**: if we are unable to make a judgement of Yes or No based on the available information.
Could the reference standard, its conduct, or its interpretation have introduced bias?	If only one signalling question was answered No or Unclear, we discussed further before making the risk of bias judgement for the domain.<br>**Low**: if all signalling questions were answered Yes.<br>**High**: if all or most signalling questions were answered No.<br>**Unclear**: if all or most signalling questions were answered Unclear.
Are there concerns that the target condition as defined by the reference standard does not match the question?	**Low**: if the study performed speciation.<br>**High**: if the study did not speciate mycobacteria isolated in culture.<br>**Unclear**: if we were unable to make a judgement of Low or High concern based on the available information.
**3B. Reference standard – COMPOSITE REFERENCE STANDARD**	
3.1B Is the reference standard likely to correctly classify the target condition?	The purpose of a composite reference standard is to diagnose people in whom TB was not detected by culture. The composite reference standard could be defined differently in each study. We identified people as having TB regardless of the criteria used in a study; study-specific criteria for defining TB were accepted. A key criterion is the initiation of TB treatment following a diagnosis of TB.<br>For composite reference standard, always judge as "Unclear".<br>**No:** if the study did not use culture methods or a clinical composite reference standard.<br>**Unclear**: If we were unable to make a judgement of Yes or No. For children, also if studies used only one specimen.
3.2B Were the reference standard results interpreted without knowledge of results of the index test?	**Yes**: if the reference standard was performed at a separate laboratory and/or performed by different people.<br>**No**: if the study stated that the reference standard result was interpreted with knowledge of the index test result.<br>**Unclear**: If we were unable to make a judgement of Yes or No based on the available information.
Could the reference standard, its conduct, or its interpretation have introduced bias?	If only one signalling question was answered no or unclear, we discussed further before making the risk of bias judgement for the domain.<br>**Low**: if all signalling questions were answered Yes.<br>**High**: if all or most signalling questions were answered No.<br>**Unclear**: if all or most signalling questions were answered unclear.
Are there concerns that the target condition as defined by the reference standard does not match the question?	**Low**: if treatment was initiated after diagnosing using the composite reference standard<br>**High**: if treatment was not initiated after using the CRS

Signalling question	Rationale for judgement
	**Unclear**: if we are unable to make a judgement of Low or High concern based on the available information.
**4. Flow and timing**	
4.1 Was there an appropriate interval between the index test and the reference standard?	**Yes**: if the index test and reference standard were performed at the same time, or if the time interval was less than or equal to seven days.<br>**No**: if the time interval was greater than seven days<br>**Unclear**: If we are unable to make a judgement of Yes or No based on the available information.
4.2 Did all patients receive the same reference standard?	**Yes**: for extrapulmonary TB if all participants in the study or a subset of participants in the study (for whom we extracted data) received the acceptable reference standard either culture or a composite reference standard. Regarding culture, we acknowledge that it is possible that some specimens could undergo solid culture and others liquid culture as the reference standard. This could potentially result in variations in accuracy, but we think the variation would be minimal.<br>**No**: if not all participants received the same reference standard<br>**Unclear**: If we are unable to make a judgement of Yes or No.
4.3 Were all patients included in the analysis?	**Yes**: if the number of participants enrolled was clearly stated and correspond to the number presented in the analysis or if exclusions were adequately described.<br>**No**: if there were participants missing or excluded from the analysis and no explanation was given.<br>**Unclear**: If we were unable to make a judgement of Yes or No based on the available information.
Could the patient flow have introduced bias?	If only one signalling question was answered No or Unclear, we discussed further before making the risk of bias judgement for the domain.<br>**Low**: if all signalling questions were answered Yes.<br>**High**: if all or most signalling questions were answered No.<br>**Unclear**: if all or most signalling questions were answered Unclear.

Appendix for QUADAS-2

WHO Standard Operating Procedures for Specimen Processing, Lymph node and other tissue, sterile specimen

1. Cut the tissue specimen into small pieces in a sterile mortar.

2. Add approximately 2 mL of sterile phosphate buffered saline (PBS).

3. Grind solution of tissue and PBS until homogeneous suspension has been obtained.

4. Place approximately 0.7 mL of the homogenized tissue in a sterile, conical screw-capped tube.

5. Double volume of specimen with TB- LAMP Sample Reagent (1.4 mL Sample Reagent to 0.7 mL of homogenized tissue).

6. Shake tube vigorously 10 to 20 times or vortex for at least 10 seconds.

7. Incubate specimen for 10 minutes at room temperature, and again shake specimen 10–20 times or vortex for at least 10 seconds.

8. Incubate specimen at room temp. for an additional 5 minutes.

9. Load into TB- LAMP as per manufacturer's instructions

WHO Standard Operating Procedures for CSF

If there is more than 5 mL of CSF available for testing:

1. Transfer all the CSF specimen to a conical centrifuge tube and concentrate the specimen at 3000 x g for 15 minutes.

2. Resuspend the pellet to a final volume of 2 mL by adding TB- LAMP sample reagent.

3. Load into TB- LAMP as per manufacturer's instructions

If there is 1–5 mL of CSF available:

1. Add an equal volume of Sample Reagent to the CSF.

2. Mix the specimen and the Sample Reagent by vortexing as described above. After 7-8 minutes at room temp., vortex the sample as above a second time.

3. Incubate for an additional 7-8 minutes (15 minutes total incubation) at room temp

4. Load the sample into the GeneXpert instrument following the manufacturer's instructions.

Appendix 6. Additional details on statistical analysis

In fitting the bivariate random effects models to obtain summary sensitivities and specificities, we carefully examined the parameter estimates—particularly the variances of the random effects and the covariance—because these are typically more uncertain than the primary model parameters (logit sensitivity and logit specificity). All analyses converged and the estimates were stable.

The traditional univariate I^2 statistic is not recommended for use in diagnostic test accuracy reviews. As outlined in the Cochrane Handbook for Systematic Reviews of Diagnostic Test Accuracy, the bivariate model provides numerical estimates of the random-effects terms that quantify heterogeneity in sensitivity and specificity. However, these estimates are not easily interpretable, as they represent variation on the log-odds scale, and are therefore typically not reported. Instead, graphical methods offer a clearer means of assessing heterogeneity. In particular, the inclusion of a 95% prediction region around the summary point estimate provides a visual indication of the likely range for sensitivity and specificity in future studies. This region incorporates the variance of the random effects for logit-transformed sensitivity and specificity, as well as their covariance.

To facilitate decision making during the WHO Guideline Development Group meeting, the performance of LC-mNAATs in key subgroups was investigated. We therefore performed subgroup analyses rather than meta-regression to investigate heterogeneity and formally compare differences in sensitivity and specificity between subgroups.

Appendix 7 . Excluded studies with reason for exclusion

S. No.	Author & Year	Reason for Exclusion
1	Aryan 2010	Case-control study
2	Aryan 2013	In-house
3	Aryan 2014	Conference abstract
4	Aryan 2016	Conference abstract
5	Baikunje 2019	Case-control study
6	Balne 2013	Case-control study
7	Balne 2015a	Reference standard not satisfied
8	Balne 2015b	Conference abstract
9	Benellam 2022	Reference standard not satisfied
10	Bentaleb 2016	In-house
11	Bhirud 2017	Unknown age group
12	Boehme 2007	Sample-wise analysis
13	Bumbrah 2023	Wrong article type
14	Cao 2015	Case-control study
15	Ckumdee 2016	Wrong target condition
16	Dayal 2020	In-house
17	Deng 2019	Wrong article type
18	Deng 2021	Case-control study
19	Dolker 2012	In-house
20	Donfack 2024	Wrong population (children)
21	Fan 2022	Data not available separately for adults and children
22	Fujisaki 2004	Not a DTA study
23	Geojith 2011	Reference standard not satisfied
24	George 2011	In-house
25	Ghosh 2017	Unknown age group
26	Govindan 2021	In-house
27	Habeenzu 2017	In-house
28	Habiburrahma 2021	Wrong article type
29	Han 2020	No 2x2 data available
30	Ignatov 2014	Not a DTA study
31	Iwamoto 2003	Not a DTA study
32	Jana 2019	Conference abstract
33	Jaroenram 2020	Not a DTA study
34	Jekloh 2022	In-house
35	Joon 2015	In-house
36	Joon 2017	In-house
37	Joon 2019	In-house
38	Juliasih 2020	Reference standard not satisfied
39	Kaewphinit 2017	In-house
40	Khan 2021	Case-control study
41	Khumwan 2022	Reference standard not satisfied
42	Kim 2023	Reference standard not satisfied
43	Kohan 2011	In-house
44	Kohan 2012	In-house

45	Kumar 2014	In-house
46	Kumar 2016	Duplicate
47	Lee 2009	Not a DTA study
48	Lee 2010	Not a DTA study
49	Li 2014a	Not a DTA study
50	Li 2014b	In-house
51	Lin 2021	Unknown age group
52	Lin 2022	Data not available separately for adults and children
53	Ling 2008	Wrong article type
54	Lisdawati 2012	Not a DTA study
55	Liu 2022	Data not available separately for adults and children
56	Miller 2013	Conference abstract
57	Mishra 2018	Data not available separately for adults and children
58	Mitha 2020	No 2x2 data available
59	Modi 2016	Case-control study
60	Moon 2015	In-house
61	Mor 2022	In-house
62	Mougang 2021	Ongoing trial
63	Nagai 2016a	Reference standard not satisfied
64	Nagai 2016b	Wrong article type
65	Nagdev 2011	Case-control study
66	Neshani 2023	Not a DTA study
67	Ngando 2017	Conference abstract
68	Nguyen 2018	Data not available separately for adults and children
69	Nimesh 2014	In-house
70	Nischal 2019	Conference abstract
71	Pandey 2008	Case-Control study
72	Pandya 2018	Ongoing trial
73	Perera 2018	In-house
74	Phetsuksiri 2019	Not a DTA study
75	Phetsuksiri 2020a	In-house
76	Phetsuksiri 2020b	In-house
77	Poudel 2009	Case-control study
78	Promsena 2018	Ongoing trial
79	Rafati 2014	In-house
80	Rajput 2019	Data not available separately for adults and children
81	Seki 2015	Conference abstract
82	Sethi 2012	Conference abstract
83	Sethi 2013a	In-house
84	Sethi 2013b	Wrong article type
85	Sethi 2013c	Duplicate
86	Sethi 2016a	In-house
87	Sethi 2016b	Duplicate
88	Sharma 2014	Case-control study
89	Sharma 2015a	Wrong article type
90	Sharma 2015b	Case-control study
91	Sharma 2016a	Case-control study

92	Sharma 2016b	Case-control study
93	Sharma 2019	Case-control study
94	Singh 2019b	Conference abstract
95	Sharma 2020a	Conference abstract
96	Sharma 2020b	Case-control study
97	Sharma 2020c	Case-control study
98	Sharma 2020d	Case-control study
99	Sharma 2020e	Case-control study
100	Sharma 2023	Reference standard not satisfied
101	Shete 2019	Wrong article type
102	Singh 2019a	Conference abstract
103	Song 2021	Reference standard not satisfied
104	Sreedeep 2020	In-house
105	Sun 2017	Case-control study
106	Teramoto 2011	Conference abstract
107	Thapa 2019	In-house
108	Thomas 2022	Conference abstract
109	Toonkomdang 2020	In-house
110	Vaidya 2017	Conference abstract
111	Wang 2021	Case-control study
112	Wen 2023	Not a DTA study
113	Wu 2017	Case-Control study
114	Wu Dan-Dan 2018	In-house
115	Yadav 2020	No 2x2 data available
116	Yadav 2023	Case-control study
117	Yan 2016	Wrong article type
118	Yang 2011	Case-control study
119	Yu 2018	Wrong article type
120	Yuan 2014	Not a DTA study
121	Zhao 2017	Case-control study
122	Zhu 2009	Not a DTA study

S. No.	References
1	Aryan E, Makvandi M, Farajzadeh A, Huygen K, Bifani P, Mousavi SL, et al. A novel and more sensitive loop-mediated isothermal amplification assay targeting IS6110 for detection of Mycobacterium tuberculosis complex. Microbiological Research. 2010 Mar 31;165(3):211–20.
2	Aryan E, Makvandi M, Farajzadeh A, Huygen K, Alvandi AH, Gouya MM, et al. Clinical value of IS6110-based loop-mediated isothermal amplification for detection of Mycobacterium tuberculosis complex in respiratory specimens. The Journal of Infection. 2013 Jun 1;66(6):487–93.
3	Aryan E, Meshkat Z, Mirbagheri SZ, Alvandi AH, Safdari H, Riahi B, et al. Improved detection of mycobacterium tuberculosis using an inhibitor-tolerant loop-mediated isothermal amplification. European Respiratory Journal. 2014 Sep 1;44(Suppl 58).
4	Aryan E, Neshani A, Sadeghian H, Safdari H. Visual Detection of Mycobacterium Tuberculosis by loop-mediated Isothermal Amplification. European Respiratory Journal. 2016 Sep 1;48(suppl 60).
5	Baikunje N, Behera D, Rajwanshi A, Sharma M, Sharma A, Sharma K. Comparative evaluation of loop-mediated isothermal amplification (LAMP) assay, GeneXpert MTB/Rif and multiplex PCR for the diagnosis of tubercular lymphadenitis in HIV-infected patients of North India. Molecular and Cellular Probes. 2019 Dec;48:101459.

6	Balne PK, Barik MR, Sharma S, Basu S. Development of a Loop-Mediated Isothermal Amplification Assay Targeting the *mpb64* Gene for Diagnosis of Intraocular Tuberculosis. Journal of Clinical Microbiology. 2013 Nov 1;51(11):3839–40.
7	Balne P, Basu S, Rath S, Barik MR, Sharma S. Loop mediated isothermal amplification assay using hydroxy naphthol blue, conventional polymerase chain reaction and real-time PCR in the diagnosis of intraocular tuberculosis. Indian Journal of Medical Microbiology. 2015 Oct 1;33(4):568–71.
8	Balne PK, Basu S, Sharma S. Loop-Mediated Isothermal Amplification for Rapid Diagnosis of Tubercular Uveitis. JAMA Ophthalmology. 2015 Feb 1;133(2):225.
9	Benellam YKJ, Ozkan O, Saadoon Aziz Z. Diagnosis of Mycobacterium tuberculosis using GeneXpert MTB / RIF and TB-LAMP techniques from pulmonary and extra-pulmonary TB patients in Iraq. Bionatura. 2022 May 15;7(2):1–8.
10	Bentaleb EM, Abid M, El Messaoudi MD, Lakssir B, Ressami EM, Amzazi S, et al. Development and evaluation of an in-house single step loop-mediated isothermal amplification (SS-LAMP) assay for the detection of Mycobacterium tuberculosis complex in sputum samples from Moroccan patients. BMC Infectious Diseases. 2016 Sep 27;16(1).
11	Bhirud P, Joshi A, Hirani N, Chowdhary A. Rapid laboratory diagnosis of pulmonary tuberculosis. International Journal of Mycobacteriology. 2017;6(3):296.
12	Boehme CC, Nabeta P, Henostroza G, Raqib R, Rahim Z, Gerhardt M, et al. Operational Feasibility of Using Loop-Mediated Isothermal Amplification for Diagnosis of Pulmonary Tuberculosis in Microscopy Centers of Developing Countries. Journal of Clinical Microbiology. 2007 Jun;45(6):1936–40.
13	Bumbrah GS, Jain S, Fatima Z, Hameed S. Efficacy of LAMP assay for Mycobacterial spp. detection to prevent treatment delays and onset of drug resistance: a systematic review and meta-analysis. Drug Target Insights. 2023 Jun 7;17:78–89.
14	Cao D, Hu L, Lin M, Li M, Ye Z, Sun H, et al. Real-time fluorescence Loop-Mediated Isothermal Amplification (LAMP) for rapid and reliable diagnosis of pulmonary tuberculosis. Journal of Microbiological Methods. 2015 Feb 1;109:74–8.
15	Ckumdee J, Kaewphinit T, Chansiri K, Santiwatanakul S. Development of Au-Nanoprobes Combined with Loop-Mediated Isothermal Amplification for Detection of Isoniazid Resistance in *Mycobacterium tuberculosis*. Journal of Chemistry. 2016 Jan 1;2016:1–8.
16	Dayal R, Yadav A, Agarwal D, Kumar M, Kamal R, Singh D, et al. Comparison of Diagnostic Yield of Tuberculosis Loop-Mediated Isothermal Amplification Assay With Cartridge-Based Nucleic Acid Amplification Test, Acid-Fast Bacilli Microscopy, and Mycobacteria Growth Indicator Tube Culture in Children With Pulmonary Tuberculosis. Journal of the Pediatric Infectious Diseases Society. 2020 Mar 10;10(2):83–7.
17	Deng S, Sun Y, Xia H, Liu Z, Gao L, Yang J, et al. Accuracy of Commercial Molecular Diagnostics for the Detection of Pulmonary Tuberculosis in China: A Systematic Review. Scientific Reports. 2019 Mar 14;9(1).
18	Deng Y, Duan Y, Gao S, Wang J. Comparison of LAMP, GeneXpert, Mycobacterial Culture, Smear Microscopy, TSPOT.TB, TBAg/PHA Ratio for Diagnosis of Pulmonary Tuberculosis. Current Medical Science. 2021 Sep 2;41(5):1023–8.
19	Dolker S, Verma R, Prakash C, Shamal A. Application of loop mediated isothermal amplification (LAMP) assay as an alternative diagnostic test for rapid tuberculosis diagnosis in limited resource setting. The Indian Journal of Animal Sciences. 2012 Nov 20;82(11):1293–7.
20	Danfack VD et al. Performance Evaluation of TB-LAMP in children. 2024.
21	Fan L, Guan B, Cheng M, Liu C, Tian Y, Li R, et al. A Comprehensive Evaluation of a Loop-Mediated Isothermal Amplification Assay for the Diagnosis of Pulmonary Tuberculosis in Children Using Bronchoalveolar Lavage Fluid. Infection and Drug Resistance. 2022 Mar;15:975–87.
22	Fujisaki R. Development of rapid molecular diagnostic method for tuberculosis based on loopmediated isothermal amplification. Teikyo Medical Journal. 2004 Jul 1;27:297–305.
23	Geojith G, Dhanasekaran S, Chandran SP, Kenneth J. Efficacy of loop mediated isothermal amplification (LAMP) assay for the laboratory identification of Mycobacterium tuberculosis isolates in a resource limited setting. Journal of Microbiological Methods. 2011 Jan;84(1):71–3.
24	George G, Mony P, Kenneth J. Comparison of the Efficacies of Loop-Mediated Isothermal Amplification, Fluorescence Smear Microscopy and Culture for the Diagnosis of Tuberculosis. Xu S, editor. PLoS ONE. 2011 Jun 17;6(6):e21007.

25	Ghosh P, Chakraborty B, Maiti P, Ray R. Comparative evaluation of loop-mediated isothermal amplification and conventional methods to diagnose extrapulmonary tuberculosis. Annals of Tropical Medicine and Public Health. 2017;10(1):160.
26	Govindan S A, Yadav R, Vaidya PC, Sethi S, Mehra N, Ranjan Das R, et al. Comparison of performances of loop-mediated isothermal amplification, XPERT MTB/RIF and BACTEC MGIT in the diagnosis of childhood tuberculosis. Journal of Paediatrics and Child Health. 2021 Mar 15;57(6):847–53.
27	Habeenzu C, Nakajima C, Solo E, Bwalya P, Kajino K, Miller M, et al. Evaluation of in-house loop-mediated isothermal amplification for tuberculosis diagnosis compared with Xpert MTB/RIF. Journal of infection in developing countries. 2017 Jun 27;11(06):440–4.
28	Habiburrahman M, Ariq H, Diah Handayani RR. Combining LAMP and Au-Nanoprobe to detect INH-RIF resistance accurately in tuberculosis: an evidence-based review. Journal of Infection in Developing Countries. 2021 Nov 30;15(11):1555–68.
29	Han M, Xiao H, Yan L. Diagnostic performance of nucleic acid tests in tuberculous pleurisy. BMC Infectious Diseases. 2020 Mar 24;20(1).
30	Ignatov KB, Barsova EV, Fradkov AF, Blagodatskikh KA, Kramarova TV, Kramarov VM. A strong strand displacement activity of thermostable DNA polymerase markedly improves the results of DNA amplification. BioTechniques. 2014 Aug 1;57(2):81–7.
31	Iwamoto T, Sonobe T, Hayashi K. Loop-Mediated Isothermal Amplification for Direct Detection of Mycobacterium tuberculosis Complex, M. avium, and M. intracellulare in Sputum Samples. Journal of Clinical Microbiology. 2003 Jun 1;41(6):2616–22.
32	Jana K, Kaminedi KVVV. Role of Loop Mediated Isothermal Amplification Assay in Detecting Mycobacterium Tuberculosis. Americna Journal of Respiratory and Critical Care Medicine. 2019 May 1;
33	Jaroenram W, Kampeera J, Arunrut N, Sirithammajak S, Jaitrong S, Boonnak K, et al. Ultrasensitive detection of Mycobacterium tuberculosis by a rapid and specific probe-triggered one-step, simultaneous DNA hybridization and isothermal amplification combined with a lateral flow dipstick. Scientific Reports. 2020 Oct 12;10(1).
34	Jekloh N, Keawliam P, Mukem D, Rudeeaneksin J, Srisungngam S, Bunchoo S, et al. Evaluation of an in-house loop-mediated isothermal amplification for Mycobacterium tuberculosis detection in a remote reference laboratory, Thailand. Revista do Instituto de Medicina Tropical de São Paulo. 2022 Jan 1;64:e57.
35	Joon D, Nimesh M, Saluja D. Loop-mediated isothermal amplification as alternative to PCR for the diagnosis of extra-pulmonary tuberculosis. International Journal of Tuberculosis and Lung Disease. 2015 Aug 1;19(8):986–91.
36	Joon D, Nimesh M, Varma-Basil M, Saluja D. Evaluation of improved IS 6110 LAMP assay for diagnosis of pulmonary and extra pulmonary tuberculosis. Journal of Microbiological Methods. 2017 Aug;139:87–91.
37	Joon D, Nimesh M, Gupta S, Kumar C, Varma-Basil M, Saluja D. Development and evaluation of rapid and specific sdaA LAMP-LFD assay with Xpert MTB/RIF assay for diagnosis of tuberculosis. Journal of Microbiological Methods. 2019 Apr 1;159:161–6.
38	Juliasih NN, Das RK, Mertaniasih NM, Neupane P, Sari RM. Comparative Evaluation of Microscopy and Loop-Mediated Isothermal Amplification (Lamp) Assay for the Diagnosis of Tuberculosis. Indian Journal of Forensic Medicine & Toxicology. 2020 Jul 30;14(3):2168–73.
39	Kaewphinit T, Ckumdee J, Chansiri K, Santiwatanakul S. Development and Evaluation of a Loop-mediated Isothermal Amplification Combined with Au-nanoprobe Assay for Rapid Detection of Mycobacterium tuberculosis. Indian Journal of Medical Microbiology. 2017 Apr;35(2):302–4.
40	Khan A, Kamra E, Singh R, Sharma V, Singh V, Mor P, et al. Diagnosis of osteoarticular tuberculosis: multi-targeted loop-mediated isothermal amplification assay versus multiplex PCR. Future Microbiology. 2021 Sep 1;16(13):935–48.
41	Khumwan P, Pengpanich S, Kampeera J, Kamsong W, Karuwan C, Sappat A, et al. Identification of S315T mutation in katG gene using probe-free exclusive mismatch primers for a rapid diagnosis of isoniazid-resistant Mycobacterium tuberculosis by real-time loop-mediated isothermal amplification. Microchemical Journal. 2022 Apr 1;175:107–8.
42	Kim HN, Lee J, Yoon SY, Jang WS, Lim CS. Rapid Detection of Mycobacterium Tuberculosis Using a Novel Point-of-Care BZ TB/NTM NALF Assay: Integrating LAMP and LFIA Technologies. Diagnostics (Basel). 2023 Apr 21;13(8):1497–7.
43	Kohan L, Shahhosseiny MH, Razavi MR, Parivar K, Moslemi E, Werngren J. Evaluation of loop mediated isothermal amplification for diagnosis of Mycobacterium tuberculosis complex in clinical samples. African Journal of Biotechnology. 2011;10(26):5096–101.

44	Kohan E, Shahhosseiny MH, Razavi MR, Parivar K, Moslemi E. Rapid detection of Mycobacterium tuberculosis complex by loop mediated isothermal amplification. Medical Science Journal of Islamic Azad Univesity - Tehran Medical Branch. 2012 Jun 10;22(2):137–42.
45	Kumar P, Pandya D, Singh N, Behera D, Aggarwal P, Singh S. Loop-mediated isothermal amplification assay for rapid and sensitive diagnosis of tuberculosis. Journal of Infection. 2014 Dec 1;69(6):607–15.
46	Kumar et al. A novel loop mediated amplification assay for rapid detection and differentiation of mycobacterium tubeculosis, m.avium complex and other mycobacterium species directly from clinical sample.
47	Lee MF, Chen YH, Peng CF. Evaluation of reverse transcription loop-mediated isothermal amplification in conjunction with ELISA–hybridization assay for molecular detection of Mycobacterium tuberculosis. Journal of Microbiological Methods. 2009 Feb;76(2):174–80.
48	Lee MF, Chen YH, Hsu HJ, Peng CF. One-tube loop-mediated isothermal amplification combined with restriction endonuclease digestion and ELISA for colorimetric detection of resistance to isoniazid, ethambutol and streptomycin in Mycobacterium tuberculosis isolates. Journal of Microbiological Methods. 2010 Oct 1;83(1):53–8.
49	Li D, Zhao J, Nie X, Wan T, Xu W, Zhao Y. Protein-coding housekeeping gene Rv2461c can be used as an amplification target in loop-mediated isothermal amplification assay for the detection of Mycobacterium tuberculosis in sputum samples. International Journal of Clinical and Experimental Pathology. 2014;7(12):8706–14.
50	Li Y, Shi L, Pan A, Cao W, Chen X, Meng H, et al. Evaluation of real-time loop-mediated isothermal amplification (RealAmp) for rapid detection of Mycobacterium tuberculosis from sputum samples. Journal of Microbiological Methods. 2014 Sep;104:55–8.
51	Lin J, Xia L, Liu X, Wei J, Xi X, Huang W, et al. Evaluation of loop-mediated isothermal amplification for the diagnosis of tuberculosis. Fudan University Journal of Medical Sciences. 2021 Jan;48(01):104–10.
52	Lin JJ, Xi XH, Xia L, Tan YJ, Chen Y, Di HQ, et al. et al. Diagnostic accuracy of loop-mediated isothermal amplification for pulmonary tuberculosis in China. Frontiers in tropical diseases. 2022 Dec 9;3.
53	Ling DI, Flores LL, Riley LW, Pai M. Commercial Nucleic-Acid Amplification Tests for Diagnosis of Pulmonary Tuberculosis in Respiratory Specimens: Meta-Analysis and Meta-Regression. PLoS ONE. 2008 Feb 6;3(2):e1536.
54	Lisdawati V, Oshibe T, Tsuji H, Sudiro TM, Adianti M, Sukarso T, et al. et al. Evaluating the use of loop-mediated isothermal amplification (LAMP) method for detection of Mycobacterium tuberculosis in Indonesian clinical isolates. Medical Journal of Indonesia. 2012 Nov 1;21(4):188–8.
55	Liu C, Fan L, Zhang J, Hong Q, Ren Y, Tian H, et al. Performance of TB-LAMP in the Diagnosis of Tuberculous Empyema Using Samples Obtained From Pleural Decortication. Frontiers in medicine. 2022 Jun 29;9:879772.
56	Miller M, Habeenzu C, Solo E, Bwalya P, Katemangwe P, Kasakwa K, et al. Latest results of ongoing evaluation of the loop-mediated isothermal amplification (LAMP) assay for the diagnosis of tuberculosis in University Teaching Hospital (UTH), Zambia. Tropical Medicine and International Health. 2013 Sep;18(suppl 1):169.
57	Mishra B, Hallur V, Behera B, Preetam C, Mishra PN, Turuk J, et al. Evaluation of loop mediated isothermal amplification (LAMP) assay in the diagnosis of tubercular lymphadenitis: A pilot study. Indian Journal of Tuberculosis. 2018 Jan;65(1):76–9.
58	Mitha M, Pillay M, Moodley JY, Balakrishna Y, Abbai N, Bhagwan S, et al. Laboratory diagnosis of tuberculous meningitis in human immunodeficiency virus–seropositive patients: Correlation with the uniform case definition. Southern African Journal of Infectious Diseases. 2020 Aug 26;35(1).
59	Modi M, Sharma K, Sharma M, Sharma A, Sharma N, Sharma S, et al. Multitargeted loop-mediated isothermal amplification for rapid diagnosis of tuberculous meningitis. The International Journal of Tuberculosis and Lung Disease. 2016 May 1;20(5):625–30.
60	Moon SH, Kim EJ, Tomono J, Miyamoto S, Mitarai S, Kim DW, et al. Detection of Mycobacterium tuberculosis complex in sputum specimens using a loop-mediated isothermal amplification assay in Korea. Journal of Medical Microbiology. 2015 Nov 1;64(11):1335–40.
61	Mor P, Sheoran A, Dahiya B, Parshad S, Nain R, Khan A, et al. Diagnosis of abdominal tuberculosis by multi-targeted (mpt64 and IS 6110) loop-mediated isothermal amplification assay. Journal of Gastroenterology and Hepatology. 2022 Nov 5;37(12):2264–71.

62	Mougang YCK, Palombi L, University of Rome Tor Vergata. DIAGNOSIS of PULMONARY TUBERCULOSIS Through HUMAN BREATH TEST Center: Douala - CAMEROON [Internet]. clinicaltrials.gov. 2021 [cited 2024 Jun 4]. Available from: https://clinicaltrials.gov/study/NCT04682990
63	Nagai Y, Iwade Y, Nakano M, Akachi S, Kobayashi T, Nishinaka T. Rapid and simple identification of Beijing genotype strain of *Mycobacterium tuberculosis* using a loop-mediated isothermal amplification assay. Microbiology and Immunology. 2016 Jul 1;60(7):459–67.
64	Nagai K, Horita N, Yamamoto M, Tsukahara T, Nagakura H, Tashiro K, et al. Diagnostic test accuracy of loop-mediated isothermal amplification assay for Mycobacterium tuberculosis: systematic review and meta-analysis. Scientific Reports. 2016 Dec 13;6(1).
65	Nagdev KJ, Kashyap RS, Parida M, Kapgate RC, Purohit HJ, Taori GM, et al. Loop-Mediated Isothermal Amplification for Rapid and Reliable Diagnosis of Tuberculous Meningitis. Journal of Clinical Microbiology. 2011 May 1;49(5):1861–5.
66	Neshani A, Zare H, Sadeghian H, Safdari H, Riahi-Zanjani B, Aryan E. A Comparative Study on Visual Detection of Mycobacterium tuberculosis by Closed Tube Loop-Mediated Isothermal Amplification: Shedding Light on the Use of Eriochrome Black T. Diagnostics. 2023 Jan 1;13(1):155.
67	Ngando L. Evaluation of the loop mediated isothermal ampilification method on the early diagnosis of pulmonary tuberculosis in Cameroon. In: Sixty-sixth Annual Meeting of American Society of Tropical Medicine & Hygiene. Baltimore, Maryland, USA: The American Journal of Tropical Medicine and Hygiene; 2017. p. 148–8.
68	Nguyen VAT, Nguyen HV, Dinh TV, Du HH, Do CN, Marks GB, et al. Evaluation of LoopampTM MTBC detection kit for diagnosis of pulmonary tuberculosis at a peripheral laboratory in a high burden setting. Diagnostic Microbiology and Infectious Disease. 2018 Mar;90(3):190–5.
69	Nimesh M, Joon D, Varma-Basil M, Saluja D. Development and Clinical Evaluation of sdaA Loop-Mediated Isothermal Amplification Assay for Detection of Mycobacterium tuberculosis with an Approach To Prevent Carryover Contamination. Land GA, editor. Journal of Clinical Microbiology. 2014 Jul;52(7):2662–4.
70	Nischal N, Das S, Singh BK, Wig N, Soneja M, Jorwal P, et al. To Evaluate the Role of Kit-Based Loop-Mediated Isothermal Amplification (TB-LAMP) Assay in the Diagnosis of Tubercular Lymphadenitis. Open Forum Infectious Diseases. 2019 Oct 1;6(Suppl 2):S731–2.
71	Pandey BD, Poudel A, Yoda T, Tamaru A, Oda N, Fukushima Y, et al. Development of an in-house loop-mediated isothermal amplification (LAMP) assay for detection of Mycobacterium tuberculosis and evaluation in sputum samples of Nepalese patients. Journal of Medical Microbiology. 2008 Apr 1;57(4):439–43.
72	Pandya C, Shah U, Khara N, eds. Side by Side comparison and evaluation of AmpEZ Real Time MTB Test with standard cultural and molecular methods. ctri.nic.in. Published June 11, 2018. Accessed June 10, 2024. https://ctri.nic.in/Clinicaltrials/pmaindet2.php?EncHid=MTY3MTc=&Enc=&userName=CTRI/2018/06/014487
73	Perera SU, Navaratne V, Nagahawatte A, Perera J, Wijayarathna CD, Alvitigala J, et al.Validating the Loop Mediated Isothermal Amplification (LAMP) technique to detect tuberculosis in a Sri Lankan laboratory setting. Ceylon Medical Journal. 2018 Mar 31;63(1):40.
74	Phetsuksiri B, Rudeeaneksin J, Srisungngam S, Bunchoo S, Klayut W, Sangkitporn S, et al. Loop-Mediated Isothermal Amplification for Rapid Identification of *Mycobacterium tuberculosis* in Comparison with Immunochromatographic SD Bioline MPT64 Rapid® in a High Burden Setting. Japanese Journal of Infectious Diseases. 2019 Mar 31;72(2):112–4.
75	Phetsuksiri B, Klayut W, Rudeeaneksin J, Srisungngam S, Bunchoo S, Toonkomdang S, et al. The performance of an in-house loop-mediated isothermal amplification for the rapid detection of Mycobacterium tuberculosis in sputum samples in comparison with Xpert MTB/RIF, microscopy and culture. Revista Do Instituto De Medicina Tropical De São Paulo. 2020;62:e36.
76	Phetsuksiri B, Rudeeaneksin J, Srisungngam S, Bunchoo S, Klayut W, Nakajima C, et al. Comparison of Loop-Mediated Isothermal Amplification, Microscopy, Culture, and PCR for Diagnosis of Pulmonary Tuberculosis. Japanese Journal of Infectious Diseases. 2020 Jul 22;73(4):272–7.
77	Poudel A, Pandey B, Lekhak B, Rijal B, Sapkota B, Suzuki Y. Clinical profiling and use of loop-mediated isothermal amplification assay for rapid detection of *Mycobacterium tuberculosis* from sputum. Kathmandu University Medical Journal. 1970 Jan 1;7(2):109–14.

78	Promsena P, Jantarabenjakul W. Diagnostic accuracy of loop-mediated isothermal amplification (TB-LAMP) for tuberculosis in children. TCTR ID: TCTR20200414003. www.thaiclinicaltrials.org. Published April 14, 2020. Accessed June 10, 2024. https://www.thaiclinicaltrials.org/#
79	Rafati A, Gill P. Microfluidic method for rapid turbidimetric detection of the DNA of Mycobacterium tuberculosis using loop-mediated isothermal amplification in capillary tubes. Microchimica Acta. 2014 Sep 11;182(3-4):523–30.
80	Rajput R, Singh P, Sarin R, Sethi P, Sharma S. Diagnostic accuracy of loop-mediated isothermal amplification assay for extra-pulmonary tuberculosis in Indian population. Journal of Microbiological Methods. 2019 Mar;158:59–65.
81	Seki et al. Clinical Evaluation of a Loop-mediated Isothermal Amplification (LAMP) Assay Targeting the hspX Gene for Detecting the Mycobacterium tuberculosis Complex in Korea.
82	Sethi et al. Loop-mediated isothermal amplification for rapid diagnosis of pulmonary tuberculosis and its comparison with IS6110 polymerase chain reaction and conventional techniques in resource-poor setting.
83	Sethi SK, Singh S, Dhatwalia SK, Yadav R, Mewara A, Singh M, et al. Evaluation of In-House Loop-Mediated Isothermal Amplification (LAMP) Assay for Rapid Diagnosis of M. tuberculosisin Pulmonary Specimens. Journal of Clinical Laboratory Analysis. 2013 Jul;27(4):272–6.
84	Sethi SK, Singh S, Dhatwalia SK, Yadav R, Mewara A, Singh M, et al. Erratum: Evaluation of In-House Loop-Mediated Isothermal Amplification (LAMP) Assay for Rapid Diagnosis of M. tuberculosis in Pulmonary Specimens. Journal of Clinical Laboratory Analysis. 2013 Sep 13;27(5):339–9.
85	Sethi SK, Singh S, Dhatwalia SK, Yadav R, Mewara A, Singh M, et al. Evaluation of In-House Loop-Mediated Isothermal Amplification (LAMP) Assay for Rapid Diagnosis of M. tuberculosisin Pulmonary Specimens. Journal of Clinical Laboratory Analysis. 2013 Jul;27(4):272–6.
86	Sethi S, Dhaliwal L, Dey P, Kaur H, Yadav R, Sethi S. Loop-mediated isothermal amplification assay for detection of Mycobacterium tuberculosis complex in infertile women. Indian Journal of Medical Microbiology. 2016 Jul;34(3):322–7.
87	Sethi S, Dhaliwal L, Dey P, Kaur H, Yadav R, Sethi S. Loop-mediated isothermal amplification assay for detection of Mycobacterium tuberculosis complex in infertile women. Indian Journal of Medical Microbiology. 2016 Jul;34(3):322–7.
88	Sharma K, Bansal R, Sharma A, Beke N, Gupta A. Loop-Mediated Isothermal Amplification for Rapid Diagnosis of Tubercular Uveitis. JAMA Ophthalmology. 2014 Jun 1;132(6):777.
89	Sharma K, Sharma A, Gupta A. Loop-Mediated Isothermal Amplification for Rapid Diagnosis of Tubercular Uveitis—Reply. JAMA Ophthalmology. 2015 Feb 1;133(2):226.
90	Sharma K, Sharma A, Dhillon M. Multi-Targeted Loop-Mediated Isothermal Amplification for Rapid Diagnosis of Osteoarticular Tuberculosis in 60 Minutes: Experience from North India [abstract]. In: Arthritis Rheumatology. American College Rheumatology; 2015.
91	Sharma K, Sharma M, Batra N, Sharma A, Dhillon MS. Diagnostic potential of multi-targeted LAMP (loop-mediated isothermal amplification) for osteoarticular tuberculosis. Journal of Orthopaedic Research. 2016 May 24;35(2):361–5.
92	Sharma M, Sharma K, Sharma A, Gupta N, Rajwanshi A. Loop-mediated isothermal amplification (LAMP) assay for speedy diagnosis of tubercular lymphadenitis: The multi-targeted 60-minute approach. Tuberculosis. 2016 Sep;100:114–7.
93	Sharma G, Tewari R, Dhatwalia SK, Yadav R, Behera D, Sethi S. A loop-mediated isothermal amplification assay for the diagnosis of pulmonary tuberculosis. Letters in Applied Microbiology. 2019 Mar 1;68(3):219–25.
94	Singh S. Multi targeted Loop-Mediated Isothermal Amplification (LAMP) for rapid diagnosis of gastrointestinal tuberculosis in 60 minutes. In: NTCA National TB Conference 2019 [Internet]. Atlanta Georgia: National Tuberculosis Coalition of America; 2019 [cited 2024 Jun 8]. Available from: https://www.tbcontrollers.org/events/annual-tb-conference/2019-poster-session-9/
95	Sharma A, Sharma K, Modi M, Sharma A. Improving the diagnosis of extra-pulmonary tuberculosis (EPTB): experience from north India. Open Forum Infectious Diseases. 2020 Oct 1;7(Suppl 1):S808–8.
96	Sharma K, Sharma M, Modi M, Goyal M, Sharma A, Ray P. Magnetic bead flocculation test: Improving the diagnosis of tuberculous meningitis (TBM) in low-resource settings. Molecular and Cellular Probes. 2020 Oct;53:101595.

97	Sharma K, Singh S, Sharma M, Batra N, Kaur V, Modi M, et al. Multi-targeted loop mediated amplification PCR for diagnosis of extrapulmonary tuberculosis. Indian Journal of Tuberculosis. 2020 Oct;67(4):479–82.
98	Sharma M, Sinha SK, Sharma M, Singh AK, Samanta J, Sharma A, et al. Challenging diagnosis of gastrointestinal tuberculosis made simpler with multi-targeted loop-mediated isothermal amplification assay. European Journal of Gastroenterology & Hepatology. 2020 May 15;32(8):971–5.
99	Sharma M, Singh R, Sharma A, Gupta V, Sharma K. IS1081-based Multi-targeted LAMP: An Opportunity to Detect Tubercular Uveitis. Ocular Immunology and Inflammation. 2020 Aug 19;30(1):168–73.
100	Sharma N, Singh P, Malik M, Sharma S, Khayyam KU, Dewan RK, et al. Rapid and Accurate Point of Care Diagnosis Tool to Combat an Ancient Foe. Applied Microbiology: Open Access. 2023 Jun 29;9(4):1–6.
101	Shete PB, Farr K, Strnad L, Gray CM, Cattamanchi A. Diagnostic accuracy of TB-LAMP for pulmonary tuberculosis: a systematic review and meta-analysis. BMC Infectious Diseases. 2019 Mar 19;19(1).
102	Singh P, Kanade S, Nataraj G. Sensitivity and specificity of loop-mediated isothermal amplification assay for diagnosis of extra-pulmonary tuberculosis: A cross-sectional study. Tuberculosis. 2019 Sep 28;54(suppl 63):PA554.
103	Song Y, Ma Y, Liu R, Shang Y, Ma L, Huo F, et al.. Diagnostic Yield of Oral Swab Testing by TB-LAMP for Diagnosis of Pulmonary Tuberculosis. Infection and Drug Resistance. 2021 Jan 1;14:89–95.
104	Sreedeep KS, Sethi S, Yadav R, Vaidya PC, Angurana SK, Saini A, et al. Loop-mediated isothermal amplification (LAMP) in the respiratory specimens for the diagnosis of pediatric pulmonary tuberculosis: A pilot study. Journal of Infection and Chemotherapy. 2020 Aug 1;26(8):823–30.
105	Sun WW, Sun Q, Yan LP, Zhang Q. The application of IS6110-baced loop-mediated isothermal amplification (LAMP) in the early diagnosis of tuberculous meningitis. Oncotarget. 2017 Feb 25;8(34):57537–42.
106	Teramoto S, Mikami Y, Toyota E, Nakajima Y. Clinical Significance Of The Newly Developed Lamp Assay For Rapid Detection Of M. Tuberculosis Of Sputum Samples Collected From The Patients Suspected Of Pulmonary Tuberculosis. American Journal of Respiratory and Critical Care Medicine. 2011 May 1;183:A1205–5.
107	Thapa J, Maharjan B, Malla M, Fukushima Y, Poudel A, Pandey BD, et al. Direct detection of Mycobacterium tuberculosis in clinical samples by a dry methyl green loop-mediated isothermal amplification (LAMP) method. Tuberculosis. 2019 Jul 1;117:1–6.
108	Thomas RE, KV S, Edwin BT, VS A, Paul JK, DM V. Loop-mediated isothermal amplification (LAMP): An advanced molecular point-of-care technique for the detection of mycobacterium tuberculosis. Agappe. 2022 Jul 7;
109	Toonkomdang S, Phinyo P, Phetsuksiri B, Patumanond J, Rudeeaneksin J, Klayut W. Pragmatic accuracy of an in-house loop-mediated isothermal amplification (LAMP) for diagnosis of pulmonary tuberculosis in a Thai community hospital. PLOS ONE. 2020 Jul 23;15(7):e0236496–6.
110	Vaidya PC, K S S, Sethi S, Singh M, Yadav R. Loop Mediated Isothermal Amplification (LAMP) in Respiratory Specimens for the Diagnosis of Paediatric Pulmonary Tuberculosis: A Pilot Study. In: American Journal of Respiratory and Critical Care Medicine. Washington: American Thoracic Society; 2017. p. A1175.
111	Wang X, Wang G, Wang Y, Quan S, Qi H, Sun L, et al. Development and Preliminary Application of Multiplex Loop-Mediated Isothermal Amplification Coupled With Lateral Flow Biosensor for Detection of Mycobacterium tuberculosis Complex. Frontiers in cellular and infection microbiology. 2021 Apr 27;11:666492.
112	Wen XH, Han YL, Cao XS, Zhao W, Yan Z, Yan L, et al. Diagnostic value of nucleic acid amplification tests for tuberculous pleural effusion. Future microbiology. 2023 Sep 1;18(14):971–83.
113	Wu D, Kang J, Li B, Sun D. Evaluation of the RT-LAMP and LAMP methods for detection of *Mycobacterium tuberculosis*. Journal of Clinical Laboratory Analysis. 2017 Sep 22;32(4):e22326.
114	Wu DD, Li BS, Kang JW, Sun DX. Comparison on the effect of RT-LAMP and LAMP method for detection of Mycobacterium tuberculosis. Chinese Journal of Zoonoses. 2018;(12):248–54.

115	Yadav R, Daroch P, Gupta P, Agarwal P, Aggarwal AN, Sethi S. Diagnostic accuracy of TB-LAMP assay in patients with pulmonary tuberculosis–a case-control study in northern India. Pulmonology. 2020 Dec;28(6):449–53.
116	Yadav B, Sharma M, Singla N, Shree R, Goyal M, Modi T, et al. Molecular diagnosis of Tuberculous meningitis: sdaA-based multi-targeted LAMP and GeneXpert Ultra. Tuberculosis. 2023 May 1;140:102339.
117	Yan L, Xiao H, Zhang Q. Systematic review: Comparison of Xpert MTB/RIF, LAMP and SAT methods for the diagnosis of pulmonary tuberculosis. Tuberculosis. 2016 Jan;96:75–86.
118	Yang B, Wang X, Li H, Li G, Cao Z, Cheng X. Comparison of loop-mediated isothermal amplification and real-time PCR for the diagnosis of tuberculous pleurisy. Letters in Applied Microbiology. 2011 Sep 19;53(5):525–31.
119	Yu G, Shen Y, Zhong F, Ye B, Yang J, Chen G. Diagnostic accuracy of the loop-mediated isothermal amplification assay for extrapulmonary tuberculosis: A meta-analysis. Kumar S, editor. PLOS ONE. 2018 Jun 26;13(6):e0199290.
120	Yuan L, Li Y, Wang M, Ke Z, Xu W. Rapid and effective diagnosis of pulmonary tuberculosis with novel and sensitive loop-mediated isothermal amplification (LAMP) assay in clinical samples: A meta-analysis. Journal of Infection and Chemotherapy. 2014 Feb 1;20(2):86–92.
121	Zhao N, Sun D, Liu J. [Visualized detection for mycobacterium tuberculosis using loop-mediated isothermal amplification assay]. Journal of Biomedical Engineering. 2017 Feb 1;34(1):72–7.
122	Zhu RY, Zhang K, Zhao M, Liu Y, Xu Y, Ju C, et al. Use of visual loop-mediated isotheral amplification of rimM sequence for rapid detection of Mycobacterium tuberculosis and Mycobacterium bovis. Journal of Microbiological Methods. 2009 Sep 1;78(3):339–43.

Appendix 8. Figure S1: Summary receiver characteristic operating plot of LC-mNAATs for detection of pulmonary TB in adults and adolescents

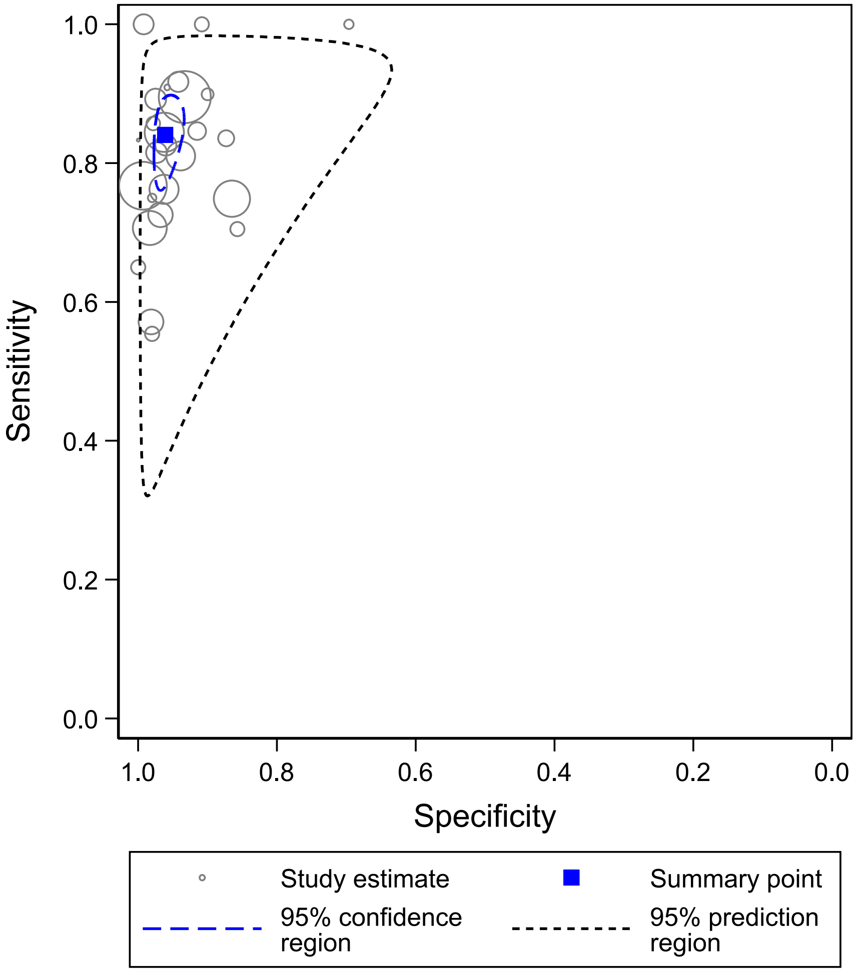

The summary point indicates the summary sensitivity and specificity and is surrounded by a 95% confidence region and a 95% prediction region. The 95% confidence region can be interpreted as a 2-dimensional confidence interval indicating the uncertainty around the summary point. The 95% prediction region provides a visual assessment of heterogeneity and represents the region within which one is 95% certain the results of a new study will.

Appendix 9. Additional forest plots

BAL = bronchoalveolar lavage; FN = false negative; FP = false positive; PLHIV = people living with HIV; PTB = pulmonary tuberculosis; TN = true negative; TP = true positive.

Study	TP	FP	FN	TN	Sensitivity (95% CI)	Specificity (95% CI)
Nakiyingi 2018	23	2	21	67	0.52 [0.37, 0.68]	0.97 [0.90, 1.00]
Spooner 2022	32	12	24	637	0.57 [0.43, 0.70]	0.98 [0.97, 0.99]
Odeume 2021	9	3	6	697	0.60 [0.32, 0.84]	1.00 [0.99, 1.00]
Nliwasa 2016	10	0	5	87	0.67 [0.38, 0.88]	1.00 [0.96, 1.00]
Danfack 2023	51	17	13	124	0.80 [0.68, 0.89]	0.88 [0.81, 0.93]
Danfack 2024b	148	53	37	810	0.80 [0.74, 0.86]	0.94 [0.92, 0.95]
N'Guessan 2016	42	8	3	3	0.93 [0.82, 0.99]	0.27 [0.06, 0.61]
Danfack 2018	36	0	0	11	1.00 [0.90, 1.00]	1.00 [0.72, 1.00]

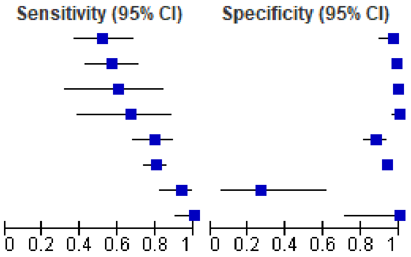

Figure S2: LC-mNAATs for detection of pulmonary TB in respiratory samples among PLHIV

Study	TP	FP	FN	TN	Sensitivity (95% CI)	Specificity (95% CI)
Nliwasa 2016	10	0	9	93	0.53 [0.29, 0.76]	1.00 [0.96, 1.00]
Odeume 2021	67	13	21	1179	0.76 [0.66, 0.85]	0.99 [0.98, 0.99]
Danfack 2023	59	2	7	81	0.89 [0.79, 0.96]	0.98 [0.92, 1.00]

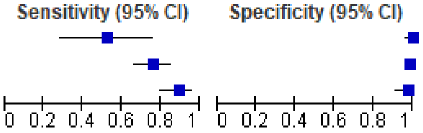

Figure S3: LC-mNAATs for detection of pulmonary TB in respiratory samples among HIV negative individuals

PLHIV, CD4 count <200

Study	TP	FP	FN	TN	Sensitivity (95% CI)	Specificity (95% CI)
Spooner 2022	14	8	9	204	0.61 [0.39, 0.80]	0.96 [0.93, 0.98]

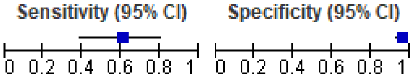

PLHIV, CD4 count ≥ 200

Study	TP	FP	FN	TN	Sensitivity (95% CI)	Specificity (95% CI)
Spooner 2022	19	2	15	419	0.56 [0.38, 0.73]	1.00 [0.98, 1.00]

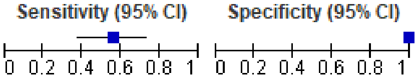

Figure S4: LC-mNAATs for detection of pulmonary TB among PLHIV by CD4 count

Study	TP	FP	FN	TN	Sensitivity (95% CI)	Specificity (95% CI)
Danfack 2018	63	1	36	2	0.64 [0.53, 0.73]	0.67 [0.09, 0.99]
Nliwasa 2016	23	0	12	0	0.66 [0.48, 0.81]	Not estimable
Spooner 2022	20	1	5	4	0.80 [0.59, 0.93]	0.80 [0.28, 0.99]
Gelaw 2017	19	0	3	1	0.86 [0.65, 0.97]	1.00 [0.03, 1.00]
Kim 2018	46	24	4	28	0.92 [0.81, 0.98]	0.54 [0.39, 0.68]
Nakiyingi 2018	35	1	3	4	0.92 [0.79, 0.98]	0.80 [0.28, 0.99]
Ou 2014	152	2	13	0	0.92 [0.87, 0.96]	0.00 [0.00, 0.84]
Pham 2018	221	1	10	1	0.96 [0.92, 0.98]	0.50 [0.01, 0.99]
Reddy 2017	47	1	2	2	0.96 [0.86, 1.00]	0.67 [0.09, 0.99]
Ou 2016	167	12	5	3	0.97 [0.93, 0.99]	0.20 [0.04, 0.48]
Gray 2016	243	0	7	0	0.97 [0.94, 0.99]	Not estimable
Danfack 2023	75	7	2	2	0.97 [0.91, 1.00]	0.22 [0.03, 0.60]
Mitarai 2011	99	3	2	11	0.98 [0.93, 1.00]	0.79 [0.49, 0.95]
N'Guessan 2016	133	10	2	2	0.99 [0.95, 1.00]	0.17 [0.02, 0.48]
Odeume 2021	69	3	1	2	0.99 [0.92, 1.00]	0.40 [0.05, 0.85]
Wang 2019	70	2	1	0	0.99 [0.92, 1.00]	0.00 [0.00, 0.84]
Bojang 2016	88	8	0	0	1.00 [0.96, 1.00]	0.00 [0.00, 0.37]
Kaku 2016	47	0	0	0	1.00 [0.92, 1.00]	Not estimable
Promsena 2022	4	0	0	0	1.00 [0.40, 1.00]	Not estimable
Yadav 2017	32	3	0	2	1.00 [0.89, 1.00]	0.40 [0.05, 0.85]
Yadav 2021	4	0	0	0	1.00 [0.40, 1.00]	Not estimable

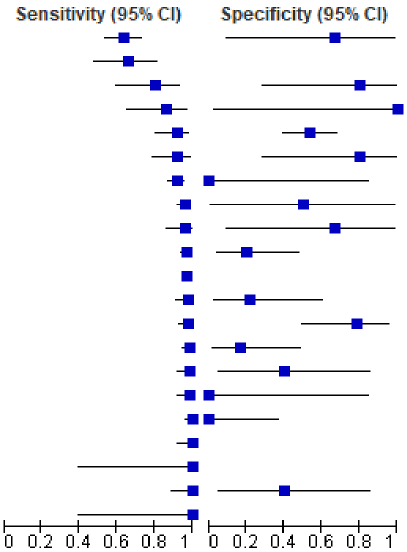

Figure S5: LC-mNAATs for detection of pulmonary TB in respiratory samples among smear positive individuals

Study	TP	FP	FN	TN	Sensitivity (95% CI)	Specificity (95% CI)
Nakiyingi 2018	11	2	34	143	0.24 [0.13, 0.40]	0.99 [0.95, 1.00]
Gelaw 2017	2	3	4	46	0.33 [0.04, 0.78]	0.94 [0.83, 0.99]
Spooner 2022	12	7	19	525	0.39 [0.22, 0.58]	0.99 [0.97, 0.99]
Getahun 2017	6	9	7	299	0.46 [0.19, 0.75]	0.97 [0.95, 0.99]
Pham 2018	71	21	81	570	0.47 [0.39, 0.55]	0.96 [0.95, 0.98]
N'Guessan 2016	11	8	11	292	0.50 [0.28, 0.72]	0.97 [0.95, 0.99]
Promsena 2022	2	0	2	8	0.50 [0.07, 0.93]	1.00 [0.63, 1.00]
Odeume 2021	30	14	29	2488	0.51 [0.37, 0.64]	0.99 [0.99, 1.00]
Ou 2014	113	14	97	938	0.54 [0.47, 0.61]	0.99 [0.98, 0.99]
Reddy 2017	33	3	26	288	0.56 [0.42, 0.69]	0.99 [0.97, 1.00]
Kaku 2016	13	3	10	136	0.57 [0.34, 0.77]	0.98 [0.94, 1.00]
Kim 2018	10	4	7	165	0.59 [0.33, 0.82]	0.98 [0.94, 0.99]
Nliwasa 2016	3	0	2	192	0.60 [0.15, 0.95]	1.00 [0.98, 1.00]
Ou 2016	155	135	103	939	0.60 [0.54, 0.66]	0.87 [0.85, 0.89]
Gray 2016	88	52	54	1333	0.62 [0.53, 0.70]	0.96 [0.95, 0.97]
Danfack 2018	62	11	34	288	0.65 [0.54, 0.74]	0.96 [0.94, 0.98]
Danfack 2023	35	12	18	203	0.66 [0.52, 0.78]	0.94 [0.90, 0.97]
Wang 2019	21	8	10	389	0.68 [0.49, 0.83]	0.98 [0.96, 0.99]
Mitarai 2011	8	9	2	25	0.80 [0.44, 0.97]	0.74 [0.56, 0.87]
Bojang 2016	9	4	0	119	1.00 [0.66, 1.00]	0.97 [0.92, 0.99]
Yadav 2017	37	0	0	379	1.00 [0.91, 1.00]	1.00 [0.99, 1.00]
Yadav 2021	1	0	0	55	1.00 [0.03, 1.00]	1.00 [0.94, 1.00]

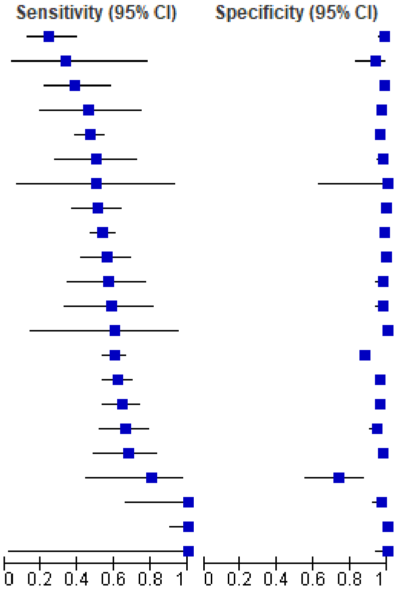

Figure S6: LC-mNAATs for detection of pulmonary TB in respiratory samples among smear negative individuals

TB-LAMP for PTB, BAL specimens

Study	TP	FP	FN	TN	Sensitivity (95% CI)	Specificity (95% CI)
Danfack 2024b	7	5	4	74	0.64 [0.31, 0.89]	0.94 [0.86, 0.98]
Promsena 2022	0	0	0	1	Not estimable	1.00 [0.03, 1.00]
Yadav 2021	1	0	0	5	1.00 [0.03, 1.00]	1.00 [0.48, 1.00]

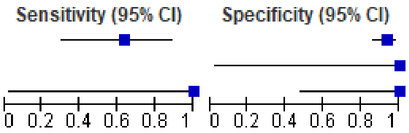

TB-LAMP for PTB, gastric aspirate specimens

Study	TP	FP	FN	TN	Sensitivity (95% CI)	Specificity (95% CI)
Promsena 2022	1	0	1	4	0.50 [0.01, 0.99]	1.00 [0.40, 1.00]
Yadav 2021	0	0	0	6	Not estimable	1.00 [0.54, 1.00]

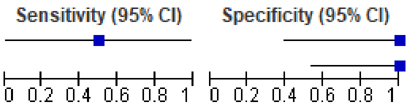

TB-LAMP for PTB, gastric lavage specimens

Study	TP	FP	FN	TN	Sensitivity (95% CI)	Specificity (95% CI)
Yadav 2021	3	1	1	19	0.75 [0.19, 0.99]	0.95 [0.75, 1.00]

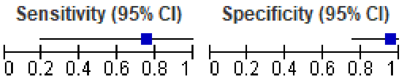

Figure S7: LC-mNAATs for detection of pulmonary TB in bronchoalveolar lavage, gastric aspirate and gastric lavage specimens

TB-LAMP for PTB, peripheral lab

Study	TP	FP	FN	TN	Sensitivity (95% CI)	Specificity (95% CI)
Nliwasa 2016	26	0	14	193	0.65 [0.48, 0.79]	1.00 [0.98, 1.00]
Ou 2014	265	16	110	938	0.71 [0.66, 0.75]	0.98 [0.97, 0.99]
Odeume 2021	99	17	30	2490	0.77 [0.68, 0.84]	0.99 [0.99, 1.00]
Cheng 2020	128	9	29	333	0.82 [0.75, 0.87]	0.97 [0.95, 0.99]
Danfack 2018	171	12	36	290	0.83 [0.77, 0.88]	0.96 [0.93, 0.98]
Gray 2016	331	52	61	1333	0.84 [0.80, 0.88]	0.96 [0.95, 0.97]
Wang 2019	91	10	11	389	0.89 [0.82, 0.94]	0.97 [0.95, 0.99]

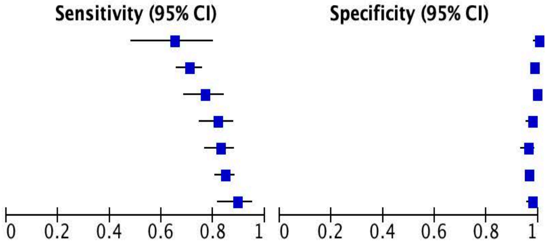

TB-LAMP for PTB, central lab

Study	TP	FP	FN	TN	Sensitivity (95% CI)	Specificity (95% CI)
Spooner 2022	32	12	24	637	0.57 [0.43, 0.70]	0.98 [0.97, 0.99]
Ren 2023	141	4	59	24	0.70 [0.64, 0.77]	0.86 [0.67, 0.96]
Reddy 2017	119	17	45	514	0.73 [0.65, 0.79]	0.97 [0.95, 0.98]
Ou 2016	322	147	108	942	0.75 [0.71, 0.79]	0.87 [0.84, 0.88]
Gelaw 2017	21	1	7	49	0.75 [0.55, 0.89]	0.98 [0.89, 1.00]
Promsena 2022	6	0	2	8	0.75 [0.35, 0.97]	1.00 [0.63, 1.00]
Pham 2018	292	22	91	571	0.76 [0.72, 0.80]	0.96 [0.94, 0.98]
Danfack 2024b	148	53	37	810	0.80 [0.74, 0.86]	0.94 [0.92, 0.95]
Kim 2018	56	28	11	193	0.84 [0.73, 0.92]	0.87 [0.82, 0.91]
Danfack 2023	110	19	20	205	0.85 [0.77, 0.90]	0.92 [0.87, 0.95]
Kaku 2016	60	3	10	136	0.86 [0.75, 0.93]	0.98 [0.94, 1.00]
Yadav 2021	15	2	2	41	0.88 [0.64, 0.99]	0.95 [0.84, 0.99]
Ou 2019	376	181	44	2519	0.90 [0.86, 0.92]	0.93 [0.92, 0.94]
Mitarai 2011	107	4	12	36	0.90 [0.83, 0.95]	0.90 [0.76, 0.97]
N'Guessan 2016	144	18	13	294	0.92 [0.86, 0.96]	0.94 [0.91, 0.97]
Wahid 2020	42	17	0	39	1.00 [0.92, 1.00]	0.70 [0.56, 0.81]
Bojang 2016	97	12	0	119	1.00 [0.96, 1.00]	0.91 [0.85, 0.95]
Yadav 2017	69	3	0	381	1.00 [0.95, 1.00]	0.99 [0.98, 1.00]

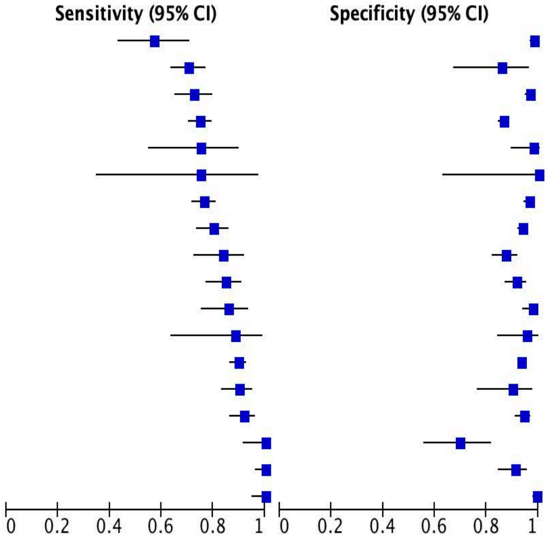

Figure S8: LC-mNAATs for pulmonary TB by lab setting

Study	TP	FP	FN	TN	Sensitivity (95% CI)	Specificity (95% CI)
Nakiyingi 2018	46	3	37	147	0.55 [0.44, 0.66]	0.98 [0.94, 1.00]
Spooner 2022	32	12	24	637	0.57 [0.43, 0.70]	0.98 [0.97, 0.99]
Ren 2023	141	4	59	24	0.70 [0.64, 0.77]	0.86 [0.67, 0.96]
Ou 2014	265	16	110	938	0.71 [0.66, 0.75]	0.98 [0.97, 0.99]
Reddy 2017	119	17	45	514	0.73 [0.65, 0.79]	0.97 [0.95, 0.98]
Ou 2016	322	147	108	942	0.75 [0.71, 0.79]	0.87 [0.84, 0.88]
Gelaw 2017	21	1	7	49	0.75 [0.55, 0.89]	0.98 [0.89, 1.00]
Promsena 2022	6	0	2	8	0.75 [0.35, 0.97]	1.00 [0.63, 1.00]
Odeume 2021	99	17	30	2490	0.77 [0.68, 0.84]	0.99 [0.99, 1.00]
Kim 2018	56	28	11	193	0.84 [0.73, 0.92]	0.87 [0.82, 0.91]
Yadav 2021	15	2	2	41	0.88 [0.64, 0.99]	0.95 [0.84, 0.99]
Wang 2019	91	10	11	389	0.89 [0.82, 0.94]	0.97 [0.95, 0.99]
Ou 2019	376	181	44	2519	0.90 [0.86, 0.92]	0.93 [0.92, 0.94]
Wahid 2020	42	17	0	39	1.00 [0.92, 1.00]	0.70 [0.56, 0.81]
Yadav 2017	69	3	0	381	1.00 [0.95, 1.00]	0.99 [0.98, 1.00]

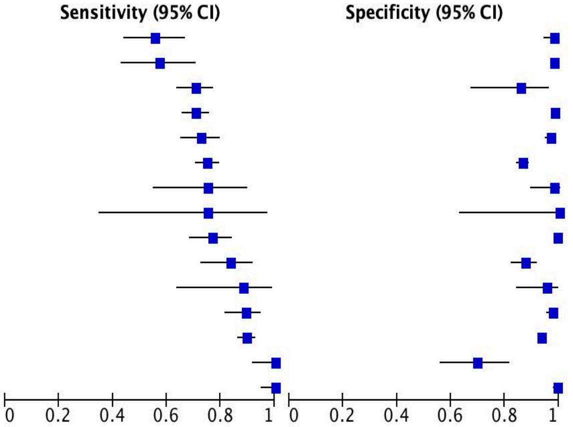

Figure S9: LC-mNAATs for pulmonary TB in high burden setting

Study	TP	FP	FN	TN	Sensitivity (95% CI)	Specificity (95% CI)
Danfack 2024a	0	2	2	61	0.00 [0.00, 0.84]	0.97 [0.89, 1.00]
Singh 2021	1	0	0	4	1.00 [0.03, 1.00]	1.00 [0.40, 1.00]

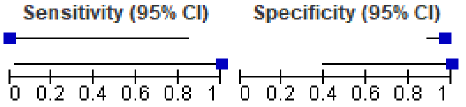

Figure S10: LC-mNAATs for detection of TB meningitis in cerebrospinal fluid

Study	TP	FP	FN	TN	Sensitivity (95% CI)	Specificity (95% CI)
Danfack 2024a	1	2	5	39	0.17 [0.00, 0.64]	0.95 [0.83, 0.99]
Singh 2021	2	1	0	17	1.00 [0.16, 1.00]	0.94 [0.73, 1.00]

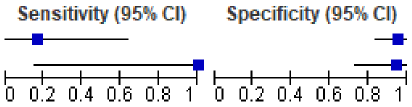

Figure S11: LC-mNAATs for detection of abdominal TB in ascitic fluid

TB-LAMP for pleural TB in pleural fluid

Study	TP	FP	FN	TN	Sensitivity (95% CI)	Specificity (95% CI)
Danfack 2024a	14	8	15	220	0.48 [0.29, 0.67]	0.96 [0.93, 0.98]
Singh 2021	6	3	2	24	0.75 [0.35, 0.97]	0.89 [0.71, 0.98]

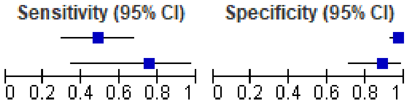

TB-LAMP for pleural TB in pleural biopsy

Study	TP	FP	FN	TN	Sensitivity (95% CI)	Specificity (95% CI)
Danfack 2024a	0	22	34	312	0.00 [0.00, 0.10]	0.93 [0.90, 0.96]

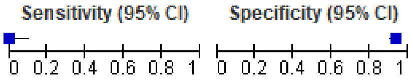

TB-LAMP for pleural TB in pleural pus

Study	TP	FP	FN	TN	Sensitivity (95% CI)	Specificity (95% CI)
Danfack 2024a	3	1	0	33	1.00 [0.29, 1.00]	0.97 [0.85, 1.00]

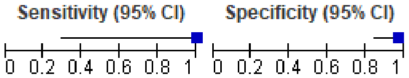

Figure S12: LC-mNAATs for detection of bone or joint TB in pleural fluid

Study	TP	FP	FN	TN	Sensitivity (95% CI)	Specificity (95% CI)
Danfack 2024a	1	0	0	4	1.00 [0.03, 1.00]	1.00 [0.40, 1.00]

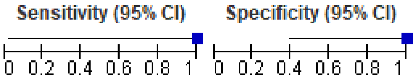

Figure S13: LC-mNAATs for detection of bone or joint TB in synovial fluid

Study	TP	FP	FN	TN	Sensitivity (95% CI)	Specificity (95% CI)
Danfack 2024a	1	0	1	30	0.50 [0.01, 0.99]	1.00 [0.88, 1.00]

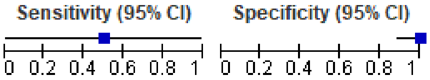

Figure S14: LC-mNAATs for detection of genitourinary TB in urine

Appendix 10. GRADE summary of findings tables

Table S1: GRADE summary of findings for LC-mNAAT on respiratory samples for detection of pulmonary TB in adults and adolescents with signs and symptoms of pulmonary TB

Population: adults and adolescents (≥10 years) with signs and symptoms of pulmonary TB

Index test: LC-mNAAT (TB-LAMP)

Reference standard: solid or liquid culture

Summary sensitivity: 0·84 (95% CI: 0·78 to 0·89) **Summary specificity**: 0·96 (95% CI: 0·94 to 0·97)

Test result	Number of results per 1,000 patients tested (95% CI)			Number of participants (studies)	Certainty of the Evidence (GRADE)
	Prevalence 2·5%	Prevalence 10%	Prevalence 30%		
True positives	**21** (20 to 22)	**84** (78 to 89)	**252** (234 to 267)	4108 (26)	⊕⊕⊕⊕ **High**
False negatives	**4** (3 to 5)	**16** (11 to 22)	**48** (33 to 66)		
True negatives	**936** (917 to 946)	**864** (846 to 873)	**672** (658 to 679)	14189 (26)	⊕⊕⊕⊕ **High**
False positives	**39** (29 to 58)	**36** (27 to 54)	**28** (21 to 42)		

CI: confidence interval

Table S2: GRADE summary of findings for LC-mNAAT on respiratory samples for detection of pulmonary TB among PLHIV

Population: PLHIV (≥10 years) with signs and symptoms of pulmonary TB

Index test: LC-mNAAT (TB-LAMP)

Reference standard: solid or liquid culture

Summary sensitivity:0·77 (95% CI: 0·61 to 0·88) **Summary specificity**:0·96 (95% CI: 0·85 to 0·99)

Test result	Number of results per 1,000 patients tested (95% CI)			Number of participants (studies)	Certainty of the Evidence (GRADE)
	Prevalence 2·5%	Prevalence20%	Prevalence 40%		
True positives	**19** (15 to 22)	**154** (122 to 176)	**308** (244 to 352)	460 (8)	⊕⊕◯◯ **Low**[a,b]
False negatives	**6** (3 to 10)	**46** (24 to 78)	**92** (48 to 156)		
True negatives	**936** (829 to 965)	**768** (680 to 792)	**576** (510 to 594)	2531 (8)	⊕⊕⊕◯ **Moderate**[c,d]
False positives	**39** (10 to 146)	**32** (8 to 120)	**24** (6 to 90)		

CI: confidence interval

Explanations

a. Point estimates of the individual studies ranged from 52% to 100% and 95% CIs did not overlap for a few studies. We could not explain this inconsistency. We downgraded one level for serious inconsistency.

b. For a prevalence of value 20%, the 95% CI around true positives and false negatives would probably lead to different decisions depending on which limits are assumed. 95% CI width of false negativity rate was 27 percentage points. We downgraded one level for serious imprecision.

c. The point estimates of individual studies ranged from 27% to 100% and 95% CIs did not overlap for a few studies. We downgraded one level for inconsistency.

d. The 95% CI was wide (0·85 to 0·99) for summary specificity. The number of participants contributing to the estimation of specificity was large (2531 participants). This was a judgement, and we did not downgrade for imprecision.

Table S3: GRADE summary of findings for LC-mNAAT on respiratory samples for detection of pulmonary among PLHIV with CD4 <200

Population: PLHIV (≥10 years) with CD4 <200 with signs and symptoms of pulmonary TB

Index test: LC-mNAAT (TB-LAMP)

Reference standard: solid or liquid culture

Single study sensitivity: 0·61 (95% CI: 0·39 to 0·80) **Single study specificity**:0·96 (95% CI: 0·93 to 0·98)

Test result	Number of results per 1,000 patients tested (95% CI)			Number of participants (studies)	Certainty of the Evidence (GRADE)
	Prevalence 5%	Prevalence 20%	Prevalence 40%		
True positives	**31** (20 to 40)	**122** (78 to 160)	**244** (156 to 320)	23 (1)	⊕○○○ **Very low**[a,b,c]
False negatives	**19** (10 to 30)	**78** (40 to 122)	**156** (80 to 244)		
True negatives	**912** (884 to 931)	**768** (744 to 784)	**576** (558 to 588)	212 (1)	⊕⊕⊕○ **Moderate**[a]
False positives	**38** (19 to 66)	**32** (16 to 56)	**24** (12 to 42)		

CI: confidence interval

Explanations

a. One study contributed data for this analysis. The study was done in South Africa, a high tuberculosis burden country. We downgraded by one level due to applicability concerns in other settings.

b. Wide 95% CI (39%- 80%). We downgraded by one level for inconsistency.

c. One study contributed very few cases to sensitivity estimation. We downgraded one level for imprecision.

Table S4: GRADE summary of findings for LC-mNAAT on lymph node tissue for detection of lymph node TB in adults and adolescents with signs and symptoms of lymph node TB

Population: adults and adolescents (≥10 years) with signs and symptoms of lymph node TB

Index test: LC-mNAAT (TB-LAMP)

Reference standard: solid or liquid culture

Summary sensitivity:0·94 (95% CI: 0·80 to 0·99) **Summary specificity**:0·90 (95% CI: 0·80 to 0·95)

Test result	Number of results per 1,000 patients tested (95% CI)			Number of participants (studies)	Certainty of the Evidence (GRADE)
	Prevalence 2·5%	Prevalence 10%	Prevalence 20%		
True positives	**24** (20 to 25)	**94** (80 to 99)	**188** (160 to 198)	35 (3)	⊕⊕◯◯ **Low**[a]
False negatives	**1** (0 to 5)	**6** (1 to 20)	**12** (2 to 40)		
True negatives	**878** (780 to 926)	**810** (720 to 855)	**720** (640 to 760)	60 (3)	⊕⊕◯◯ **Low**[b]
False positives	**97** (49 to 195)	**90** (45 to 180)	**80** (40 to 160)		

CI: confidence interval

Explanations

a. There were very few cases (35) with lymph node TB contributing to sensitivity estimation. For a prevalence value of 10%, we thought the 95% CI around true positives and false negatives may lead to different decisions depending on which confidence limits are assumed. We downgraded two levels for very serious imprecision.

b. Very few participants (60) contributed to specificity estimation. For a prevalence value of 10%, we thought the very wide 95% CI around true negatives and false positives may lead to different decisions depending on which confidence limits are assumed. We downgraded two levels for very serious imprecision.

Table S5: GRADE summary of findings for LC-mNAAT on CSF for detection of TB meningitis in adults and adolescents with signs and symptoms of TB meningitis

Patient or population: adults and adolescents (≥10 years) with signs and symptoms of TB meningitis

Index test: LC-mNAAT (TB-LAMP)

Reference standard: solid or liquid culture

Range of sensitivities:0·01 to 0·9 **Range of specificities**:0·97 to 0·99

Test result	Number of results per 1,000 patients tested (Ranges)			Number of participants (studies)	Certainty of the Evidence (GRADE)
	Prevalence2·5%	Prevalence10%	Prevalence20%		
True positives	0 to 25	1 to 99	2 to 198	3 (2)	⊕◯◯◯ Very low[a,b]
False negatives	0 to 25	1 to 99	2 to 198		
True negatives	946 to 965	873 to 891	776 to 792	67 (2)	⊕⊕◯◯ Low[c]
False positives	10 to 29	9 to 27	8 to 24		

CI: confidence interval

Explanations

a. The sensitivity ranged from 0-100% across three studies. We could not explain this variation. We downgraded one level for inconsistency.

b. There were too few cases (three) with tuberculous meningitis from two studies. We downgraded two levels for very serious imprecision.

c. Limited sample size from two studies. One study with five participants had wide 95% CI for specificity (40%-100%). We downgraded two levels for very serious imprecision
